# Transcriptomic analysis of *Asparagus officinalis* cultivars with varying levels of freezing tolerance over fall acclimation and spring deacclimation periods

**DOI:** 10.3389/fpls.2024.1442784

**Published:** 2024-08-16

**Authors:** Arshdeep Singh Gill, David J. Wolyn

**Affiliations:** Department of Plant Agriculture, University of Guelph, Guelph, ON, Canada

**Keywords:** asparagus, freezing tolerance, RNA-Seq, transcriptomics, acclimation, differential gene expression

## Abstract

Asparagus (*Asparagus officinalis* L.*)* is an important vegetable crop in southern Ontario, Canada, where winter air and soil temperatures below 0°C are common. Consequently, cultivars growing in this area must possess winterhardiness and freezing tolerance for survival. Asparagus acquires freezing tolerance in the fall through cold acclimation and loses freezing tolerance in the spring through deacclimation. To understand the molecular bases of these processes, transcriptomic analysis (RNA-Seq) was conducted on two cultivars, one adapted, ‘Guelph Millennium’ (GM), and one unadapted, ‘UC157’ (UC), to the winter conditions of southern Ontario. RNA extracted from bud and rhizome tissues, sampled on three dates during early spring and late fall, was subjected to sequencing. In the fall, the numbers of differentially expressed (DE) genes at the second and third harvests increased, relative to the first harvest, in dormant buds and rhizomes as freezing tolerance of cultivars increased, and the majority of DE genes were downregulated. In spring, freezing tolerance decreased as plants deacclimated and most genes DE at second and third harvests were upregulated in both cultivars. GM had lower LT_50_ (lethal temperature at which 50% of plants die) values and hence higher freezing tolerance than UC on specific sampling dates during both spring and fall, and expression patterns of specific genes were correlated with LT_50_ differences. Functional analysis revealed that these genes were involved in carbohydrate metabolic process, plant hormone signal transduction (auxin and gibberellin), proline metabolism, biosynthesis of secondary metabolites, circadian rhythm, and late embryogenesis abundant proteins and could be associated with cold acclimation and deacclimation processes. These findings will help researchers understand the molecular mechanisms of freezing tolerance in asparagus, leading to breeding and genetic strategies to improve the trait.

## 1 Introduction

Asparagus is an herbaceous perennial vegetable crop cultivated worldwide. Southern Ontario is the major producer of asparagus in Canada where air and soil temperatures of -20°C and -5°C, respectively, are common during the winter (Weather Innovations, 2011). Consequently, asparagus cultivars must possess winterhardiness and freezing tolerance for survival. During fall, the above-ground asparagus fern turns yellow and senesces, leaving the crown to overwinter (Landry and Wolyn, 2011). To cope with freezing temperatures, plants undergo a process known as cold acclimation where exposure to low non-freezing temperatures during late summer or early fall triggers changes that increase their ability to survive (Ouellet, 2007). Genetic and molecular evidence indicates that cold acclimation is a complex phenomenon involving the alteration of several metabolic pathways with the synthesis of specific metabolites, proteins, lipids and carbohydrates, and changes in membrane composition (Fürtauer et al., 2019). In spring as temperatures rise, freezing tolerance is gradually lost via deacclimation which allows plants to transition to regular growth and development (Vyse et al., 2019).

Two asparagus cultivars, ‘Guelph Millennium’ (GM) and ‘UC157’ (UC), showed different patterns of adaptation and freezing tolerance during fall acclimation and spring deacclimation in southern Ontario (Panjtandoust and Wolyn, 2016a, b). GM, being a locally bred cultivar, is most adapted and maintains high yields for many years (Landry and Wolyn, 2011). UC was bred in California, shows poor adaptation to southern Ontario and its yield declines after 2-3 years of production (Wolyn, 2018). GM had lower LT_50_ (lethal temperature at which 50% of plants die) as compared to UC in early- and mid-October (Panjtandoust and Wolyn, 2016a) suggesting it may acclimate and achieve high freezing tolerance early in the fall. However, both cultivars obtained similar levels of freezing tolerance by late-October and early-November (Panjtandoust and Wolyn, 2016a). The enhanced freezing tolerance in GM was highly correlated with low-molecular-weight fructan, glucose, proline, and protein concentrations in rhizomes (Landry and Wolyn, 2011; Panjtandoust and Wolyn, 2016a). These substances are considered cryoprotectants as they help protect plant cells from damage caused by low temperatures (Manasa et al., 2022). In the spring, both cultivars showed no difference in LT_50_ early in the season (Panjtandoust and Wolyn, 2016b). However, UC deacclimated and lost freezing tolerance late in the season, while GM retained its freezing tolerance and showed no change in LT_50_ levels (Panjtandoust and Wolyn, 2016b). The concentrations of low-molecular-weight fructans, glucose, proline, and proteins decreased in rhizomes with deacclimation and values for GM were greater than those for UC (Panjtandoust and Wolyn, 2016b). Research on asparagus seedlings in controlled environments also supported the observation that GM exhibits higher freezing tolerance than UC, consistent with the field adaptation, and the concentrations of various metabolites correlated with the response (Kim and Wolyn, 2015). Thus, research to date has associated the increase in freezing tolerance in asparagus with timely acclimation and increased concentrations of rhizome cryoprotectants. The effects of metabolite levels on freezing tolerance in asparagus buds remains unknown.

Cold acclimation leads to large-scale transcriptome changes by affecting the expression of a large number of genes by either up- or down-regulation (Kidokoro et al., 2022). Transcriptome analysis, particularly a high throughput sequencing technology such as RNA-Seq has proven to be a valuable tool in characterizing molecular regulatory activity and studying gene expression patterns in response to cold stress and cold acclimation in various plant species (Shen et al., 2014; Zheng et al., 2015; Ma et al., 2022). In *Arabidopsis thaliana* L., 20% of 22,043 genes responded to low-temperature acclimation and of these, 514 have been identified as cold-regulated (Hannah et al., 2005) and included genes which encode osmolytes and cryoprotective proteins and other molecules that enhance freezing tolerance. Transcription factors (TFs) such as C-repeat binding factors (CBFs), *INDUCER OF CBF EXPRESSION 1*, and *MYB15* have also been shown to play important roles in *A. thaliana* cold regulation and acclimation (Nakashima et al., 2009). CBFs can recognize and bind to specific DNA sequences (cis-elements) in the promoters of cold regulated genes, triggering their expression and contributing to cold tolerance.

Metabolic pathways such as those involved in sugar, flavonoid, and amino acid metabolism have been linked to the synthesis of cryoprotectant compounds and molecules that help plants alleviate damage caused by cold stress (Kidokoro et al., 2022). Transcriptome studies in several species have revealed up- or down-regulation of numerous genes involved in several processes related to freezing tolerance such as: cell wall biosynthesis, hormone metabolism, and CBF pathways in *A. thaliana* (Liu et al., 2022), ATPase activity, epigenetics, and photosynthesis in rice (*Oryza sativa* L.*)* (Shen et al., 2014), Ca^2+^ signal transduction and CBF pathway genes in alfalfa (*Medicago sativa* L.) (Wang et al., 2022), phenylpropanoid biosynthesis, photosynthesis, starch biosynthetic pathway, ABA signaling, and various TFs such as APETALA2/Ethylene responsive factor (AP2/ERF), MYB, and WRKY in Eucalyptus (*Eucalyptus globulus* L.) (Aguayo et al., 2023), plant hormone signal transduction, starch and sucrose metabolism, peroxisome biogenesis, and photosynthesis in apple (*Malus domestica* L*.)* (Zhou et al., 2021), and starch and sucrose metabolism, cellulose degradation, MAPK signaling pathway, plant hormone signal transduction, and TFs such as AP2/ERF, basic helix-loop-helix (bHLH), and MYB in kiwifruit (*Actinidia Lindl*. L.) (Sun et al., 2021).

Transcriptome analyses in various plant species have provided valuable insights into the complex molecular mechanisms underlying freezing tolerance and response to cold stress. Most freezing tolerance studies tend to focus on aboveground tissues (leaves and buds) as it is difficult to study roots in their natural environment (Ambroise et al., 2020). However, aboveground and belowground tissues have different physiologies and stress response mechanisms (Vanwallendael et al., 2019; Ambroise et al., 2020). A significant knowledge gap exists for understanding the molecular pathways and signaling mechanisms involved in cold stress response in underground, overwintering asparagus crowns. Understanding winter hardiness mechanisms in an herbaceous perennial such as asparagus, where tissues such as buds, rhizomes, and storage roots may be affected differently, can provide significant insight for breeding and selection to improve freezing tolerance.

The objective of this study was to identify genes and pathways that show differential expression in relation to freezing tolerance in asparagus. The primary focus was to understand the molecular processes involved in fall cold acclimation and spring deacclimation. To achieve this, a comparative transcriptome analysis of two asparagus cultivars with different freezing tolerance levels was performed.

## 2 Materials and methods

### 2.1 Experimental design

Two cultivars (GM and UC) with varying levels of freezing tolerance were planted in a Renton sandy loam soil within four blocks at the Simcoe Research Station in Simcoe, ON, Canada (Lat. 42° 51′ N; Long. 80°16′W, elevation 240.5 m) in a split-split plot design on 24 June 2019. Two harvest times (fall and spring) were treated as whole/main plots. Within the whole plots, three sampling dates were randomized as sub-plots, and two cultivars were randomized within sub-plots. A similar independent experiment was planted on 16 June 2020 at a different site at the Simcoe Research Station on a Brookton sandy clay loam soil. Each experimental unit consisted of 35 plants spaced 30 cm within a row and rows were spaced 1.25 m.

### 2.2 Plant establishment

Seeds of cultivars GM and UC were obtained from Fox Seeds, Simcoe, ON, Canada and Jersey Asparagus Farms, Pittsgrove, NJ, U.S.A., respectively. These cultivars originated from crosses between two heterozygous parents, so each is a full-sib family. Seeds were planted in 288-cell plug trays using a potting mix (Sunshine LC 4, Sun Gro Horticulture Canada, Seba Beach, AB, Canada) in March 2019 and 2020. Each year, three-week-old seedlings were transplanted to 50-cell plug trays using the same potting mix as above. Plants were grown in the greenhouse at 25/20°C (day/night) under natural irradiance supplemented with a 12 h photoperiod from high-pressure sodium lamps (200 - 300 µmol m^-1^ s^-2^). Plants were fertilized weekly with 20N-3.5P-16.6K (1.25 g L^−1^; Plant Products Limited; Brampton, ON, Canada). Ten-week-old seedlings were transplanted in the field into 20 cm deep trenches in June 2019 and June 2020. Crowns were covered with 5 cm of soil, and the trenches were gradually filled during the summer. Guard rows were planted to separate sampling date treatments.

### 2.3 Field sampling

Plots planted in the summer of 2019 were harvested in the spring and fall of 2020 and those planted in the summer of 2020 were harvested in the spring and fall of 2021. Each year, crowns were harvested on three dates during late-fall and early-spring (**Supplementary Table 1**) to capture expected patterns of cold acclimation and deacclimation. For example, in the fall, the first harvest was conducted before fern yellowing for both cultivars. The second harvest date occurred when the fern of GM started yellowing while the fern of UC was mostly green. The third harvest commenced when the fern both cultivars had senesced completely. In spring, the first harvest was conducted immediately after the soil thawed when both cultivars were expected to be dormant. At the second harvest, UC was predicted to have initiated deacclimation while GM remained dormant. For the third harvest date, both cultivars were deacclimated and spears were emerging from the soil.

On each harvest date, crowns were dug manually and cleaned of soil. Of the 35 crowns harvested per experimental unit, 25 were selected randomly for LT_50_ estimation and stored in bins overnight at 4°C. The remaining 10 crowns were stored on ice and taken to the lab immediately where they were washed with water to remove soil. Buds were then separated from rhizomes and epidermal tissue was removed from buds and rhizomes. Buds and rhizomes from each plant were frozen separately in liquid nitrogen and stored at -80°C until RNA extraction.

### 2.4 LT_50_ measurements

Twenty-five crowns from each plot were trimmed such that storage roots were 20 cm in length. Crowns were planted individually into pots (17 cm × 22 cm) with bark mix (70% aged pine bark fines, 25% peat moss, 5% compost; ASB Greenworld Ltd. Mount Elgin, ON, Canada) and watered thoroughly. Five random pots from each of the four field replicates for both cultivars were distributed into each of four chest freezers, resulting in 20 pots per cultivar per freezer. The remaining five pots from each field replicate per cultivar served as controls and were stored at 4°C for 24 h, then moved to a greenhouse for regrowth at 20/15°C (day/night) under a 16 h photoperiod supplemented by high-pressure sodium lamps (200 - 300 µmol m^-1^ s^-2^). The pots in the chest freezers were chilled at 4°C for 24 h, then subjected to freezing treatments of 0, -4, -8, -12 and -16°C, achieved by decreasing the temperature at a rate of 1°C per h. After the desired temperature was achieved, it was held constant for 12 h. Then, four random pots of each cultivar were removed from each replicate freezer, and the temperature was decreased to the next treatment level. The above, freezing treatment profile was followed for plants harvested in fall 2020, fall 2021, and spring 2021, while plants harvested in spring 2020 were subjected to freezing treatments of 0, -6, -12 and -18°C, where the temperature was decreased by 3°C per h, and five random pots of each cultivar were removed from each replicate freezer after each temperature treatment. Plants were then grown for 4 weeks in a greenhouse under the same conditions as described above and rated as alive if at least one vigorous spear grew, or dead. The number of alive plants was recorded for each cultivar.

### 2.5 Statistical analysis

LT_50_ values were estimated from plant count data using Proc Probit of SAS 9.4 (RRID: SCR_008567) (SAS Institute, 2009). Analysis of residuals was performed with Proc Univariate to test normality; no transformations were necessary (SAS Institute, 2009). LT_50_ data were analyzed with restricted maximum likelihood covariance estimates using the Proc Mixed procedure, considering fixed effects of cultivar, harvest date, and cultivar × harvest date, and random effects of year, replication (year), and their interactions with fixed effects (SAS Institute, 2009). The year, year × cultivar, and year × harvest date effects were significant, so data were analyzed separately by year. The significance of LT_50_ values was declared with Tukey’s honestly significant difference test (*p*-value ≤ 0.05) using the R v4.1.2 software package (RRID: SCR_001905) (http://www.r-project.org). Soil temperature data for the Simcoe Research Station for 2020 and 2021 were obtained from Weather Innovations (https://www.weatherinnovations.com/).

### 2.6 RNA isolation and sequencing

Total RNA was extracted separately from rhizome and bud tissues of each crown using a Trizol reagent kit (Cat #15596018, Invitrogen, Ottawa, ON, Canada) as per the manufacturer’s instructions. Three μl of RNA from each of the 10 plants were pooled to make one sample per experimental unit. For the fall samplings of each replicate experiment, 36 RNA samples (three harvest dates, two cultivars, two tissues, three replications) were sent for sequencing. In the spring, two stages of bud growth (dormant and growing buds) were observed in UC at the first harvest date and in both cultivars at the second harvest date. Consequently, RNA was extracted separately from these two types of bud tissues along with rhizome tissues. In total, 45 RNA samples were submitted for sequencing in the spring for each replicate experiment.

RNA concentration was determined by using a Nano-Drop spectrophotometer ND-1000 v3.5.2 (RRID: SCR_016517) (Thermo Fisher Scientific, Waltham, MA, U.S.A.) and an Agilent 2100 Bioanalyzer system (RRID: SCR_018043) (Agilent, Santa Clara, CA, U.S.A.) was used to estimate the RNA integrity number (RIN). High-quality RNA (concentration > 1000 ng/μl, 260/280 ratio > 2, RIN > 7) was used for sequencing. RNA samples (30 μl in 1.5 ml Eppendorf tubes) were sent to ‘The Center for Applied Genomics at Sick Kids Hospital’, Toronto, ON, Canada, for sequencing. NEB (New England Biolabs Ltd.) Next Ultra II Directional polyA mRNA library prep kit (Cat #E7760L, New England Biolabs, Whitby, ON, Canada) was used to construct libraries from the mRNA. The cDNA libraries were sequenced on two lanes of an Illumina NovaSeq 6000 SP flowcell (RRID: SCR_016387) with a read length of 2x 100 bases (paired-end).

### 2.7 Quality check and alignment of RNA-Seq reads

The quality of raw sequencing data was evaluated using the FastQC (version 0.11.9) tool (RRID: SCR_014583) (Andrews, 2010). Raw untrimmed reads were mapped to the *Asparagus officinalis* (Aspof.V1) reference genome (Harkess et al., 2017) using STAR software (Spliced Transcript Alignment to a Reference, version 2.7.8a) (RRID: SCR_004463) with default parameters (Dobin et al., 2013).

### 2.8 Differential gene expression analysis

The raw read counts for each gene file (non-normalized counts) were used for differential expression analysis with the DEseq2 R package (RRID: SCR_015687) (Love et al., 2017). Four factors were used in the design formula for statistical analysis (Design = replication + harvest + cultivar + tissue). Gene expression analysis was performed using the above full model, but to identify differentially expressed (DE) genes, read counts from the second and third harvest dates were compared to the respective first harvest date during the same fall or spring season for each tissue in each cultivar. A contrast statement was used to identify DE genes for a particular comparison. To account for multiple hypotheses testing as thousands of genes were tested for significance, the *p*-value for each gene was adjusted using the Benjamini and Hochberg correction i.e., false discovery rate (FDR) (Benjamini and Hochberg, 1995). Genes having a fold change greater than 2 (log_2_fold > 2) and an adjusted *p*-value (FDR) less than or equal to 0.05, were considered as DE. For data visualization, variance stabilizing transformation was used to produce log_2_ scale data of the normalized counts obtained from DESeq2. These log transformed data were used to build principal component analysis (PCA) plots and dispersion plots using plotPCA and plotDispEst functions, respectively, of the DEseq2 package.

### 2.9 Functional annotation of genes

The putative functions of DE genes were determined using DAVID (Database for Annotation, Visualization, and Integrated Discovery) software (RRID: SCR_001881) (Huang et al., 2009; Sherman et al., 2022). A minimum count threshold (number of genes belonging to an annotation term) of two was used to find enriched categories bearing an EASE score (a modified Fisher Exact *p*-value) of less than or equal to 0.1. Enriched categories used to create heatmaps were selected after a Benjamini and Hochberg correction (adjusted *p*-value, FDR ≤ 0.1) to account for multiple comparisons. Heatmaps were built with the matrix visualization software Morpheus (RRID: SCR_017386) (https://software.broadinstitute.org/morpheus/) using the adjusted *p*-values for enriched GO terms and metabolic enriched pathways using the KEGG database. Genes DE in the various metabolic pathways and showing expression patterns consistent with LT_50_ were selected to create heatmaps for different metabolic pathways using Morpheus software.

### 2.10 Identification of transcription factors among DE genes

Protein sequences of all asparagus DE genes (https://ftp.ebi.ac.uk/ensemblgenomes/pub/release-57/plants/fasta/asparagus_officinalis/pep/) during fall acclimation or spring deacclimation were used to identify TFs using the iTak online program (Zheng et al., 2016). Heatmaps of TFs showing expression patterns consistent with LT_50_ were constructed with the matrix visualization software Morpheus (https://software.broadinstitute.org/morpheus/) using log_2_fold values.

### 2.11 Quantitative reverse transcription-PCR validation

Total RNA was extracted separately from buds and rhizome tissues of all samples used for RNA-Seq using a Trizol reagent kit as per the manufacturer’s instructions (Invitrogen, Ottawa, ON, Canada). One μg of high-quality RNA was used for first-strand cDNA synthesis with the RevertAid First Strand cDNA Synthesis Kit (Cat #K1622, ThermoFisher Scientific, Toronto, ON, Canada) according to the manufacturer’s instructions. The cDNA was diluted with RNase free water (1:40), and 4 μl of cDNA was used for qRT-PCR. Three downregulated and four upregulated genes showing expression consistent with freezing tolerance patterns were selected for qRT-PCR validation. Gene-specific primers were designed for selected genes and the *ACTIN* gene (internal control) (**Supplementary Table 2**) using the PrimerQuest tool of Integrated DNA Technologies (Coralville, IA, U.S.A.) based on the following parameters: 55-60°C melting temperature, 55-60% GC content, and a 75 - 150 bp amplified fragment length. The Primer-BLAST tool (RRID: SCR_003095) was used to check the specificity of each primer pair to the target gene. The qRT-PCR analysis was carried out on a QuantStudio 6 Flex RT-PCR system (RRID: SCR_020239) using the PowerUp SYBR Green Master Mix kit (Cat #A25743, ThermoFisher Scientific, Toronto, ON, Canada) as per the manufacturer’s instructions. Cycle threshold (Ct) values obtained from qRT-PCR were analyzed to determine the relative gene expression between harvest dates by using the comparative Ct (2−ΔΔCT) method (Livak and Schmittgen, 2001). Three technical replicates from independent tissue samples were analyzed for each of three biological replicates of each sample. First, relative gene expression was calculated, using an internal reference *ACTIN* gene, as ΔCt (target gene – *ACTIN* gene). Secondly, as in RNA-Seq, the first harvest was used as a calibrator to calculate the relative gene expression values as ΔΔCt (second or third harvest – first harvest). Log_2_fold change was estimated using the ΔΔCt values as per the 2−ΔΔCT formula (Livak and Schmittgen, 2001). Means and standard errors for relative gene expression were calculated from three biological replicates. A linear regression using the R v4.1.2 software package was performed to assess the consistency between RNA-Seq and qRT-PCR analyses.

## 3 Results

### 3.1 LT_50_ assessment

**Figure 1:**
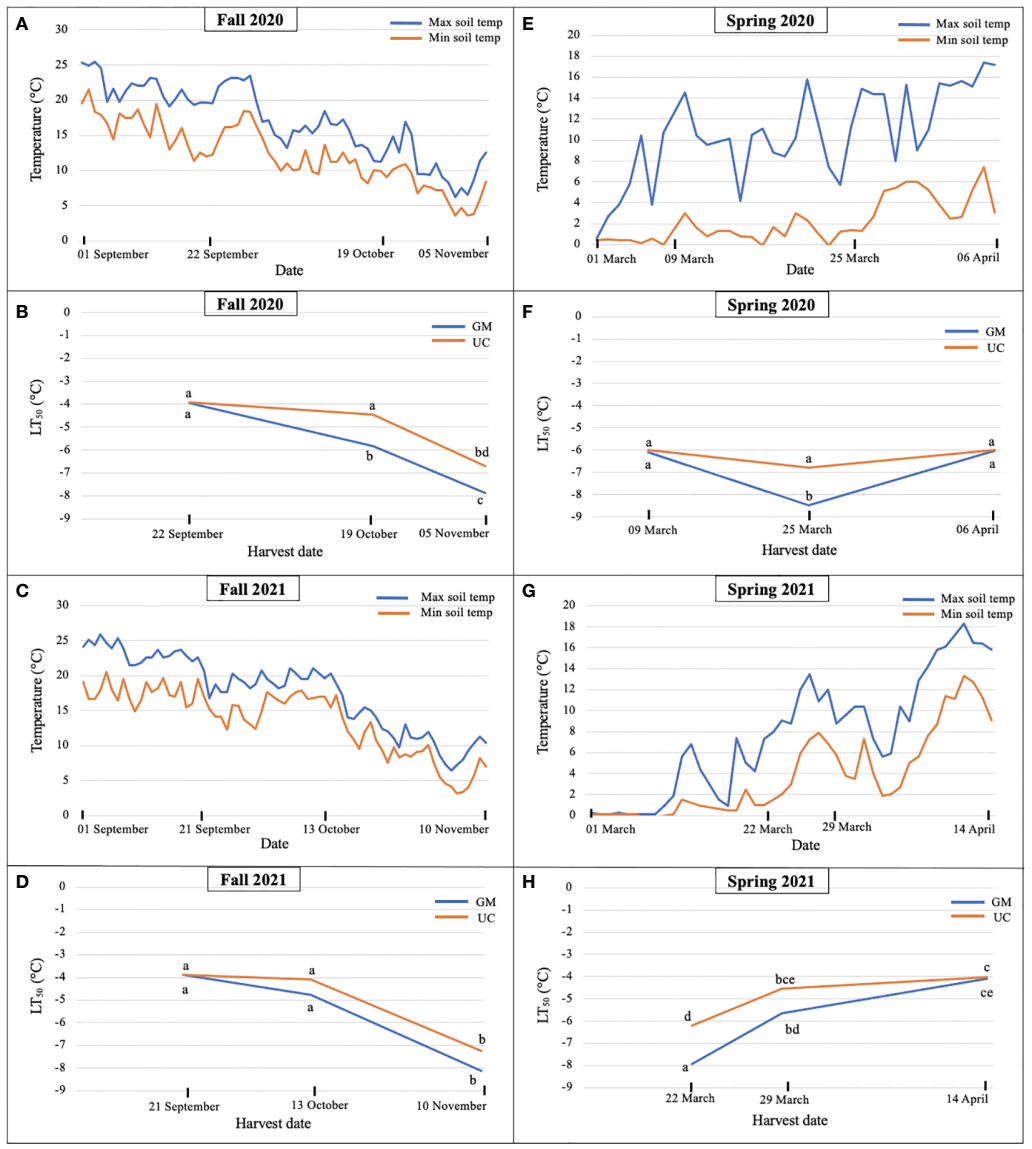
Daily maximum and minimum soil temperatures at 10 cm during **(A)** fall 2020, **(C)** fall 2021, **(E)** spring 2020, and **(G)** spring 2021 and LT_50_ (lethal temperature at which 50% plants die) values for cultivars ‘Guelph Millennium’ (GM) and ‘UC157’ (UC) in **(B)** fall 2020, **(D)** fall 2021, **(F)** spring 2020, and **(H)** spring 2021 at the Simcoe research station, Simcoe, ON, Canada. Letters represent significant differences as determined by Tukey’s honestly significant difference test (*p*-value ≤ 0.05).

In fall 2020, soil temperatures dropped from 22 September to 19 October, and continued to decrease until 05 November (**Figure 1A**). LT_50_ values for GM and UC did not differ in September, however, values for GM were lower (increased freezing tolerance) than those for UC in October and November (**Figure 1B**). LT_50_ values in GM decreased from September to November while that for UC occurred only from October to November. GM acquired freezing tolerance earlier and at greater levels than UC as the October and November values for GM and UC, respectively, did not differ and GM had lower values than UC at the November harvest date (**Figure 1B**).

In fall 2021, soil temperatures changed minimally between 21 September and 13 October but decreased thereafter by approximately 10°C (**Figure 1C**). Consistent with constant temperature, no difference in freezing tolerance or LT_50_ was observed between the first and second harvest dates for both cultivars (**Figure 1D**); differences between cultivars were also not detected. For both cultivars, LT_50_ decreased (freezing tolerance increased) during the period between 13 October and 10 November, and values of GM and UC did not differ at the November harvest date (**Figure 1D**).

In spring 2020, soil temperature increased from 09 March to 06 April with weekly fluctuations (**Figure 1E**). Cultivars did not differ for LT_50_ on 09 March and 06 April; values also did not differ over these two dates (**Figure 1F**). On 25 March, LT_50_ was lower for GM than UC (**Figure 1F**), which coincided with a rapid 10°C decrease in temperature on 23 March (**Figure 1E**). Despite no change in LT_50_ between cultivars across 09 March and 06 April, differences in bud growth were observed for harvest dates and cultivars. On 09 March, buds from GM crowns were dormant while some from UC had started growing or deacclimating. On 25 March, both dormant and growing buds were found in both cultivars and by 06 April, all buds from both cultivars were growing with some spears emerging from the ground.

In spring 2021, LT_50_ of GM and UC increased (decreased freezing tolerance) from 22 March to 14 April as soil temperatures increased (**Figures 1G, H**). Cultivars only differed on 22 March where LT_50_ was greater (lower freezing tolerance) for UC than GM. On 29 March, LT_50_ for UC appeared greater than that for GM, but the difference was not significant. Both cultivars had the same LT_50_ by 14 April (**Figure 1H**).

Overall, LT_50_ results suggested that freezing tolerance increased as soil temperatures decreased in the fall. In fall 2020, GM had a higher freezing tolerance than UC at the second and third harvests (**Figure 1B**), however, both cultivars achieved the same levels of freezing tolerance by the third harvest in fall 2021 (**Figure 1D**). In spring 2020, freezing tolerance of GM increased at the second harvest as the soil temperature decreased but decreased at the third harvest as the soil temperature increased, while UC did not show any change in freezing tolerance throughout the season (**Figure 1F**). In spring 2021, freezing tolerance decreased for both cultivars as soil temperatures increased. UC lost its freezing tolerance earlier as compared to GM but both cultivars deacclimated and lost freezing tolerance by the third harvest (**Figure 1H**).

### 3.2 RNA-Seq overview/validation

Average yields of raw paired-end reads ranged from 23 to 31.4 million in the fall and spring of 2020 and 2021 (**Table 1**). Across both seasons and years, the number obtained for individual samples ranged from 15.3 to 68.9 million (**Supplementary Tables 3–6**). For the reads obtained across both seasons and years, more than 95% had a base call accuracy of 99.9% (Q score > 30) which showed the good quality of raw data (**Table 1**). Approximately 86% of raw reads mapped uniquely to single genomic locations of the asparagus reference genome for both the fall and spring data over both years (**Table 1**). The average percentages of multi-mapped and unmapped reads ranged from 5.4 to 8.1% and 7.0 to 8.0%, respectively.

**Table 1:** Overview of the sequencing and mapping from RNA-Seq samples in asparagus.

Season	Total number of samples	Average no. of raw reads (in millions)	Average reads with Q score >30 (Percent)	Average uniquely mapped reads (Percent)	Average multi-mapped reads (Percent)	Average unmapped reads (Percent)
Fall 2020	36	24.4	95.7	85.7	7.3	7.0
Fall 2021	36	26.0	97.3	86.2	6.6	7.2
Spring 2020	45	23.0	98.5	84.4	8.1	7.6
Spring 2021	45	31.4	97.4	86.7	5.4	8.0

After filtering genes expressed at a low level (read counts less than 10), approximately 19,500 genes were retained for each of the fall and spring samples of 2020 and 2021 and were used for differential gene expression analysis. For the fall and spring seasons of both years, dispersion estimates scattered around the curve with values decreasing as the mean expression level increased, which showed that all four datasets were good fits for the DESeq2 model (**Supplementary Figure 1**).

### 3.3 Principal component analysis

**Figure 2:**
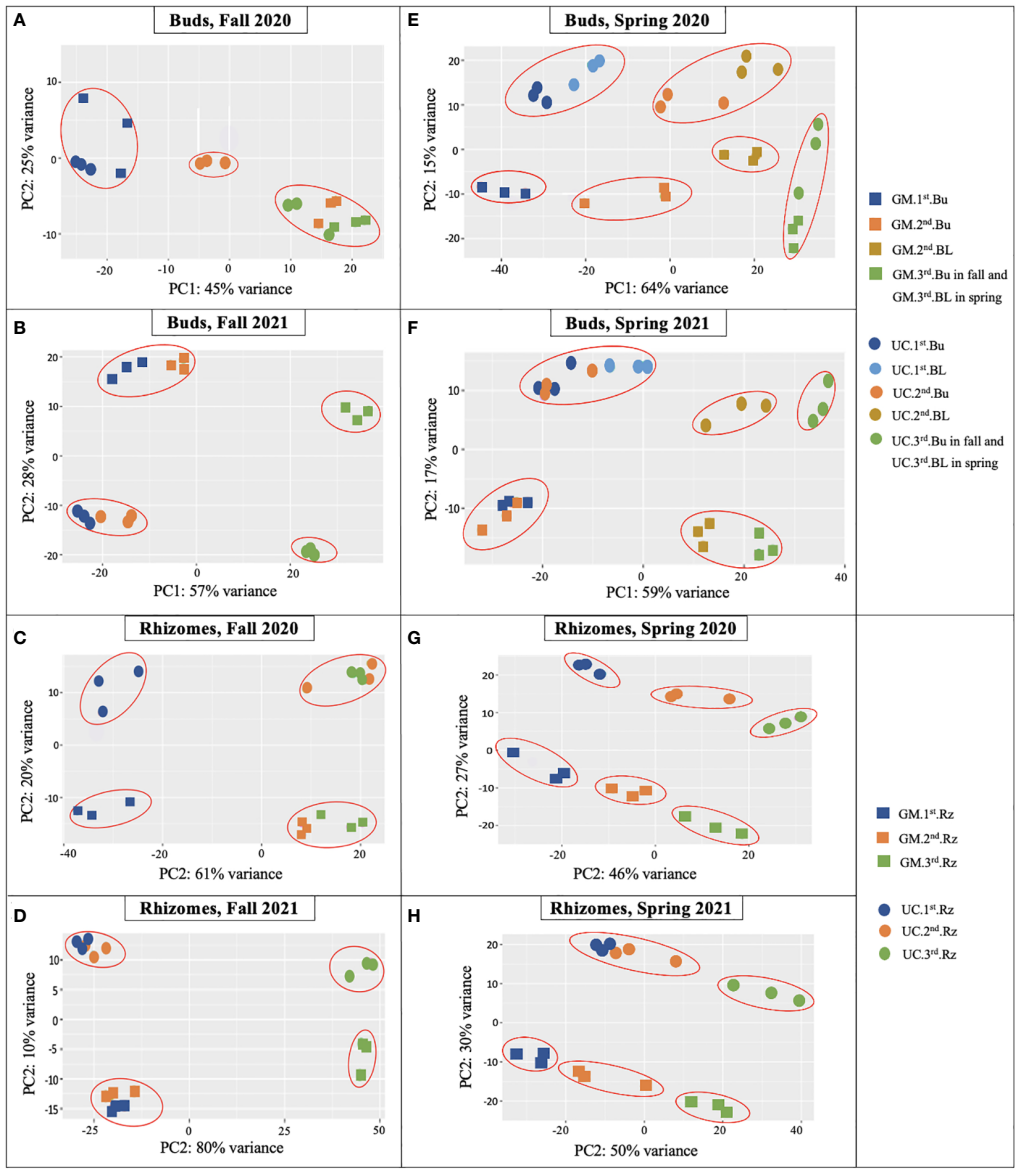
Principal component analysis (PCA) plots of RNA-Seq data from dormant buds (Bu) in **(A)** fall 2020, **(B)** fall 2021; from rhizomes (Rz) in **(C)** fall 2020, **(D)** fall 2021; from dormant and growing buds (BL) in **(E)** spring 2020, **(F)** spring 2021; and from rhizomes in **(G)** spring 2020, **(H)** spring 2021 in asparagus cultivars ‘Guelph Millennium’ (GM) and ‘UC157’ (UC). Three dots of the same color represent the three biological replicates of each harvest date. 1^st^, first harvest (22 September 2020 and 21 September 2021 in fall, 09 March 2020 and 22 March 2021 in spring); 2^nd^, second harvest (19 October 2020 and 13 October 2021 in fall, 25 March 2020 and 29 March 2021 in spring); 3^rd^, third harvest (05 November 2020 and 10 November 2021 in fall, 06 April 2020 and 14 April 2021 in spring).

PCA on bud and rhizome samples of both cultivars provided insights into the overall clustering of samples based on the expression patterns of genes. Analysis of data by season in each year generally distinguished tissue type, but not cultivar or harvest date (**Supplementary Figure 2**). Assessment of the eight combinations of season, year, and tissue type showed clustering, and consequently consistency, of the three biological replicates across cultivars and harvest dates (**Figure 2**).

For buds of the fall 2020 experiment, clear variation existed between GM and UC at the second harvest (**Figure 2A**). The second and third harvests clustered together and were clearly separated from the first harvest for GM. UC, however, showed variation among the three harvests. LT_50_ data were consistent with PCA except for decreasing LT_50_ of GM from the second and third harvest and LT_50_ cultivar differences at the third harvest (**Figure 1B**). For rhizomes during the fall of 2020, the magnitude of variance between cultivars was consistent across all harvests (**Figure 2C**). Within each cultivar, the second and third harvests grouped together and were separated from the first harvest. LT_50_ data were consistent with the cultivar differences at the second and third harvests (**Figure 1B**). Although PCA grouped the second and third harvests of each cultivar, LT_50_ decreased during this period for both GM and UC.

For buds and rhizomes of the fall 2021 experiment, cultivars separated distinctly over harvest date (**Figures 2B, D**), although LT_50_ values did not differ between cultivars (**Figure 1D**). Within each cultivar, the first and second harvests clustered together and were distinct from the third harvest in both tissues (**Figures 2B, D**), which was consistent with LT_50_ results; no differences were observed between the first and second harvests, but values for these harvests differed from those of the third harvest (**Figure 1D**).

For buds and rhizomes in spring 2020, clear separations were observed both between cultivars and among harvest dates (**Figures 2E, G**), although LT_50_ values for both cultivars did not differ between the first and third harvests, and cultivars only differed at the second harvest (**Figure 1F**). In spring 2021, gene expression variance was observed between cultivars at all harvest dates, and among harvest dates within each cultivar, for both buds and rhizome tissues (**Figures 2F, H**). These results were consistent with LT_50_ data for cultivar differences at the first harvest and increasing LT_50_ for both cultivars from the first to third harvest (**Figure 1H**).

Overall, the PCA plots showed consistency of biological replicates in the experiment and separated cultivars and harvest dates with some exceptions. LT_50_ differences between cultivars and among sampling dates were generally supported by PCA plots, with cultivars showing stronger associations than harvest dates.

### 3.4 Differential gene expression analysis

**Figure 3:**
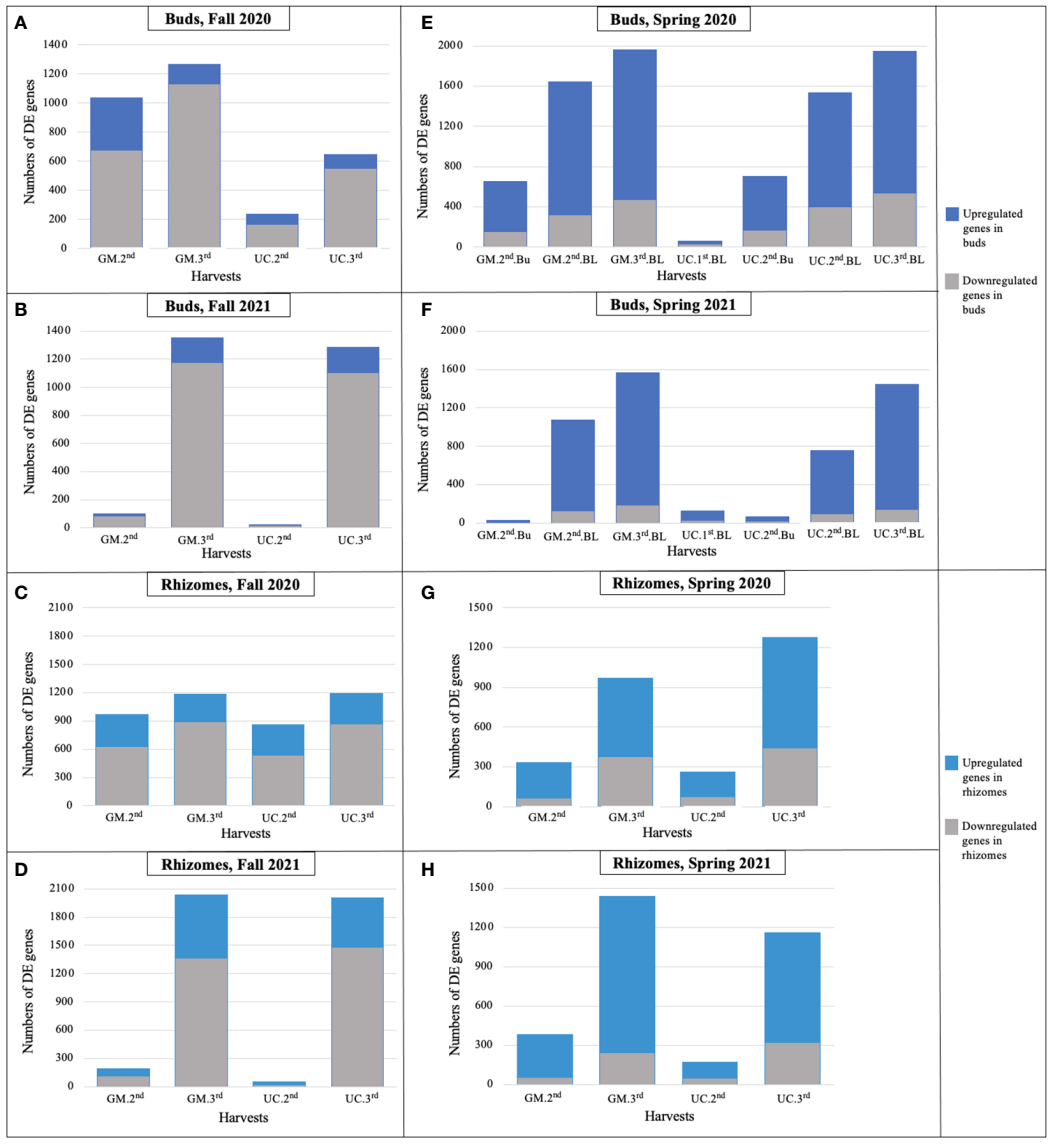
Number of differentially expressed genes for each cultivar in dormant buds (Bu) during **(A)** fall 2020, **(B)** fall 2021; in rhizomes (Rz) during **(C)** fall 2020, **(D)** fall 2021; in dormant and growing buds (BL) during **(E)** spring 2020, **(F)** spring 2021; and in rhizomes during **(G)** spring 2020, **(H)** spring 2021, relative to first harvest within each season. GM, cultivar ‘Guelph Millennium’; UC, cultivar ‘UC157’. 1^st^, first harvest (22 September 2020 and 21 September 2021 in fall, 09 March 2020 and 22 March 2021 in spring); 2^nd^, second harvest (19 October 2020 and 13 October 2021 in fall, 25 March 2020 and 29 March 2021 in spring); 3^rd^, third harvest (05 November 2020 and 10 November 2021 in fall, 06 April 2020 and 14 April 2021 in spring).

During the fall of 2020 and 2021, as soil temperatures and LT_50_ of GM and UC decreased (or freezing tolerance increased) from the first to third harvest, the number of DE genes increased in the dormant buds and rhizome tissues of both cultivars (**Figure 3**; **Table 2**), with the majority of DE genes being downregulated in both tissues (**Supplementary Table 7, 8**). In the fall of 2020, more genes were DE in dormant buds of GM than UC at the second and third harvests, which is consistent with the observation that GM had higher freezing tolerance (lower LT_50_) than UC at both harvests (**Figure 3A**; **Table 2**). However, rhizomes of both cultivars had a similar number of DE genes at the second and third harvests, despite variation for LT_50_ or freezing tolerance between cultivars (**Figure 3C**; **Table 2**). In the fall of 2021, there was little change in gene expression of GM and UC between the first and second harvests in both tissues which was consistent with no change in LT_50_ or freezing tolerance for the two cultivars during this period (**Table 2**). However, the number of DE genes significantly increased in both cultivars at the third harvest in both dormant buds and rhizome tissues (**Figures 3B, D**), and a similar number of genes showed DE between cultivars for both tissues which corresponded to the same levels of freezing tolerance (**Table 2**).

**Table 2:** Patterns of LT_50_ and gene expression among asparagus cultivars ‘Guelph Millennium’ (GM) and ‘UC157’ (UC) over the fall and spring of 2020 and 2021.

Season	Soil temperature	LT_50_			Number of DE genes in dormant buds			Number of DE genes in rhizomes		
		GM	UC	Cultivar specificity	GM	UC	Cultivar specificity	GM	UC	Cultivar specificity
Fall										
1^st^ harvest, 2020				GM = UC						
2^nd^ harvest, 2020	decrease	decrease	NC	GM < UC	increase	NC	GM > UC	increase	increase	GM = UC
3^rd^ harvest, 2020	decrease	decrease	decrease	GM < UC	increase	increase	GM > UC	increase	increase	GM = UC
1^st^ harvest, 2021				GM = UC						
2^nd^ harvest, 2021	NC	NC	NC	GM = UC	NC	NC	GM = UC	NC	NC	GM = UC
3^rd^ harvest, 2021	decrease	decrease	decrease	GM = UC	increase	increase	GM = UC	increase	increase	GM = UC
Spring										
1^st^ harvest, 2020				GM = UC						
2^nd^ harvest, 2020	increase	decrease	NC	GM < UC	increase	increase	GM = UC	increase	increase	GM = UC
3^rd^ harvest, 2020	increase	NC	NC	GM = UC	increase	increase	GM = UC	increase	increase	GM < UC
1^st^ harvest, 2021				GM < UC						
2^nd^ harvest, 2021	increase	increase	increase	GM < UC	increase	increase	GM > UC	increase	increase	GM > UC
3^rd^ harvest, 2021	increase	increase	increase	GM = UC	increase	increase	GM = UC	increase	increase	GM > UC

NC, no change. Fall Season (1^st^ harvest, 22 September 2020 and 21 September 2021; 2^nd^ harvest, 19 October 2020 and 13 October 2021; 3^rd^ harvest, 05 November 2020 and 10 November 2021). Spring season (1^st^ harvest, 09 March 2020 and 22 March 2021; 2^nd^ harvest, 25 March 2020 and 29 March 2021; 3^rd^ harvest, 06 April 2020 and 14 April 2021).

In the spring of 2020 and 2021, as the soil temperatures increased over harvest dates, the number of DE genes increased in growing buds as well as in the rhizomes of both cultivars (**Figure 3**; **Table 2**), with the majority of genes being upregulated (**Supplementary Table 9, 10**). In 2020, LT_50_ did not change from the first to third harvest in GM and UC, so the observed increase in gene expression in both cultivars during this period may not directly be related to freezing tolerance (**Figures 3E, G**; **Table 2**). However, in 2021, an increase in the numbers of DE genes in growing buds and rhizomes of both cultivars over harvest dates coincided with an increase in LT_50_ values (decrease in freezing tolerance) and LT_50_ levels were consistent with patterns of gene expression in growing buds and rhizomes (**Figures 3F, H**; **Table 2**).

Overall, in the buds and rhizomes of both cultivars, the majority of genes downregulated in the fall, as plants acclimated, were upregulated in the spring as plants deacclimated and a small number of genes upregulated in the fall were downregulated in the spring (**Table 3**, data not shown). The patterns of gene expression were consistent with the freezing tolerance (LT_50_) differences and/or similarities in the dormant buds of fall 2020, dormant buds and rhizomes of fall 2021, and growing buds and rhizomes of spring 2021. However, gene expression patterns could not be related to freezing tolerance patterns in rhizomes during fall of 2020 and in the growing buds and rhizomes during spring of 2020.

**Table 3:** Summary of genes differentially expressed (DE) in the dormant buds (Bu), growing buds (BL), and rhizomes (Rz) of asparagus cultivars, ‘Guelph Millennium’ (GM) and ‘UC157’ (UC) during fall acclimation and spring deacclimation.

DE genes	Consistent with LT_50_						Inconsistent with LT_50_					
	Down in fall		Up in spring	Up in fall		Down in spring	Down in fall		Up in spring	Up in fall		Down in spring
	Bu	Rz	BL and Rz	Bu	Rz	BL and Rz	Bu	Rz	BL and Rz	Bu	Rz	BL and Rz
Carbohydrate metabolism												
*FRUCTOSE-BISPHOSPHATE ALDOLASE (FBA*)	+		+					+				
*BETA-FRUCTOFURANOSIDASE 3*	+	+	+	+								
*GALACTINOL-SUCROSE GALACTOSYLTRANSFERASE (RFS1)*				+	+	+						
*GALACTINOL SYNTHASE 1 (GOLS1)*				+	+	+						
*BETA-GLUCOSIDASE BOGH3B*	+	+	+									
*ENDOGLUCANASE 3*	+	+	+									
*SUCROSE-PHOSPHATE SYNTHASE 2 (SPS2*)					+	+						
*TREHALOSE-PHOSPHATE PHOSPHATASE F (TPPF)*					+					+		+
*SUCROSE SYNTHASE 2 (SS2)*				+	+							+
Bidirectional sugar transporter (SWEET)	+	+	+	+	+	+						
Photosynthesis												
Chlorophyll a-b binding proteins	+		+									
Photosynthesis genes	+		+									
Plant hormone signal transduction												
Auxin binding/induced proteins	+	+	+ (BL)									
Ethylene responsive transcription factor		+	+				+					
Xyloglucan endotransglucosylases	+								+			
Gibberellin 20-oxidases	+	+	+									
Gibberellin-regulated proteins					+	+				+		
Arginine and proline metabolism												
*DELTA*-1-*PYRROLINE*-5-*CARBOXYLATE SYNTHASE (P5CS)*				+	+	+						
*PROLINE DEHYDROGENASE 2 (PRODH2)*	+	+	+									
*PROLINE TRANSPORTER 2*	+	+	+									
Circadian rhythm												
*CCA1*										+	+	
*LNK 1, LNK2*										+	+	
*TWO-COMPONENT RESPONSE REGULATOR (PRR95)*										+	+	+
Heat shock proteins (HSP) (**Protein processing in endoplasmic reticulum)**					+	+				+		
Late embryogenesis abundant (LEA) proteins				+	+	+						
Peroxidases (**Phenylpropanoid biosynthesis)**	+	+	+									
Glutathione transferases (GST) **(Glutathione metabolism)**		+	+									
Steroid biosynthesis genes	+	+	+									
Flavonoid biosynthesis		+	+									

Gene expression generalized over the second and third harvests of fall 2020 and 2021 or spring 2020 and 2021. ‘Consistent with LT_50_’ in fall indicates differential expression of genes was consistent with either greater LT_50_ values of GM than UC in fall 2020 (GM > UC) or equal LT_50_ values of both cultivars in fall 2021 (GM = UC). ‘Consistent with LT_50_’ in spring indicates differential gene expression was consistent with equal LT_50_ values at third harvests either in dormant buds or rhizomes during 2020 or 2021. Down, downregulated genes. Up, upregulated genes. + (BL), upregulated only in the growing buds.

### 3.5 Functional annotation of DE genes

**Figure 4:**
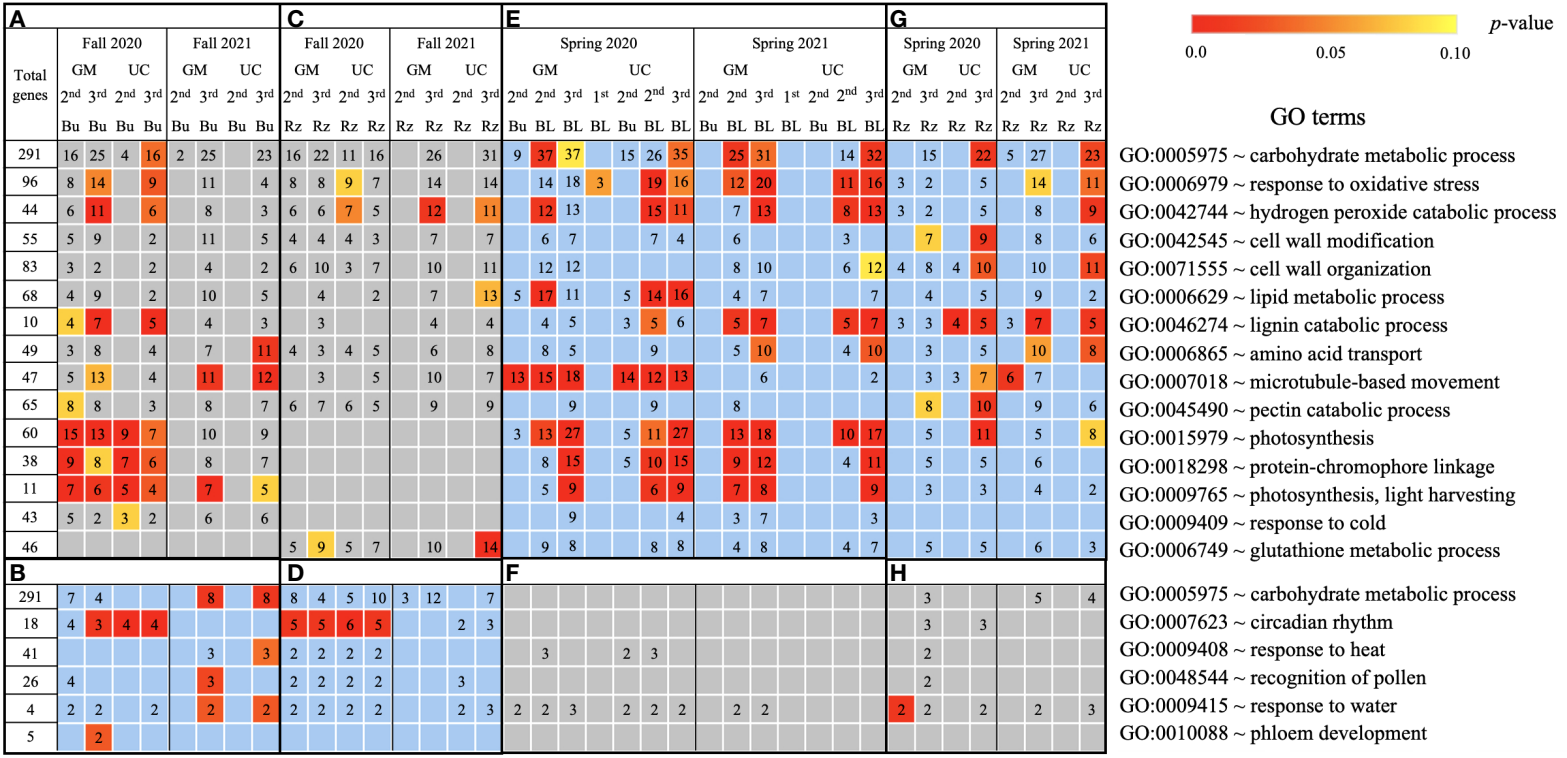
Biological process gene ontology (GO) terms assigned to genes differentially expressed relative to the first harvest for asparagus cultivars ‘Guelph Millennium’ (GM) and ‘UC157’ (UC). GO terms **(A)** downregulated and **(B)** upregulated in dormant buds (Bu) during the fall (2020 and 2021); GO terms **(C)** downregulated and **(D)** upregulated in rhizomes (Rz) during the fall (2020 and 2021); GO terms **(E)** upregulated and **(F)** downregulated in dormant and growing buds (BL) during the spring (2020 and 2021); GO terms **(G)** upregulated and **(H)** downregulated in rhizomes during the spring (2020 and 2021). The number of genes assigned to each GO term are presented in the cells; blank boxes equal zero. Within upregulated (blue area) and downregulated (grey area) genes, significantly enriched terms are color-coded based on an adjusted *p*-value i.e., false discovery rate of less than 0.1 (red most significant). 1^st^, first harvest; 2^nd^, second harvest; 3^rd^, third harvest.

**Figure 5:**
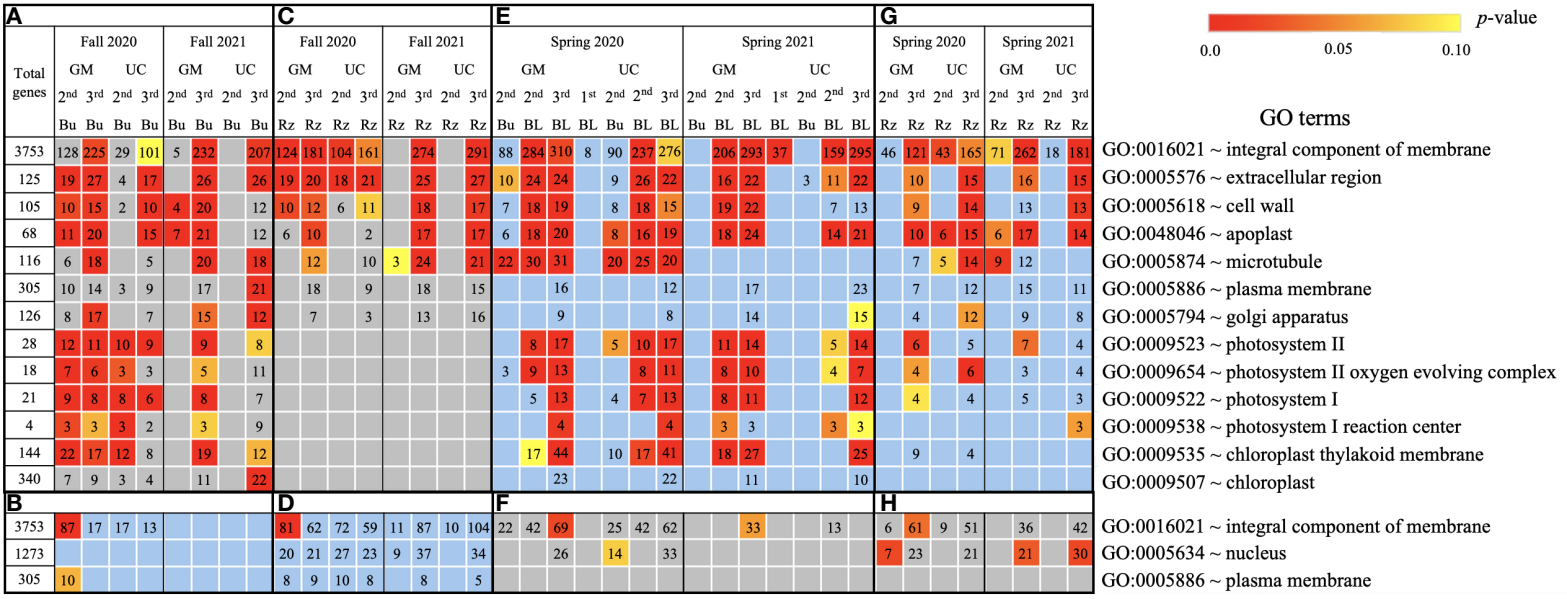
Cellular process gene ontology (GO) terms assigned to genes differentially expressed relative to the first harvest for asparagus cultivars ‘Guelph Millennium’ (GM) and ‘UC157’ (UC). GO terms **(A)** downregulated and **(B)** upregulated in dormant buds (Bu) during the fall (2020 and 2021); GO terms **(C)** downregulated and **(D)** upregulated in rhizomes (Rz) during the fall (2020 and 2021); GO terms **(E)** upregulated and **(F)** downregulated in dormant and growing buds (BL) during the spring (2020 and 2021); GO terms **(G)** upregulated and **(H)** downregulated in rhizomes during the spring (2020 and 2021). The number of genes assigned to each GO term are presented in the cells; blank boxes equal zero. Within upregulated (blue area) and downregulated (grey area) genes, significantly enriched terms are color-coded based on an adjusted *p*-value i.e., false discovery rate of less than 0.1 (red most significant). 1^st^ harvest; 2^nd^ harvest; 3^rd^ harvest.

**Figure 6:**
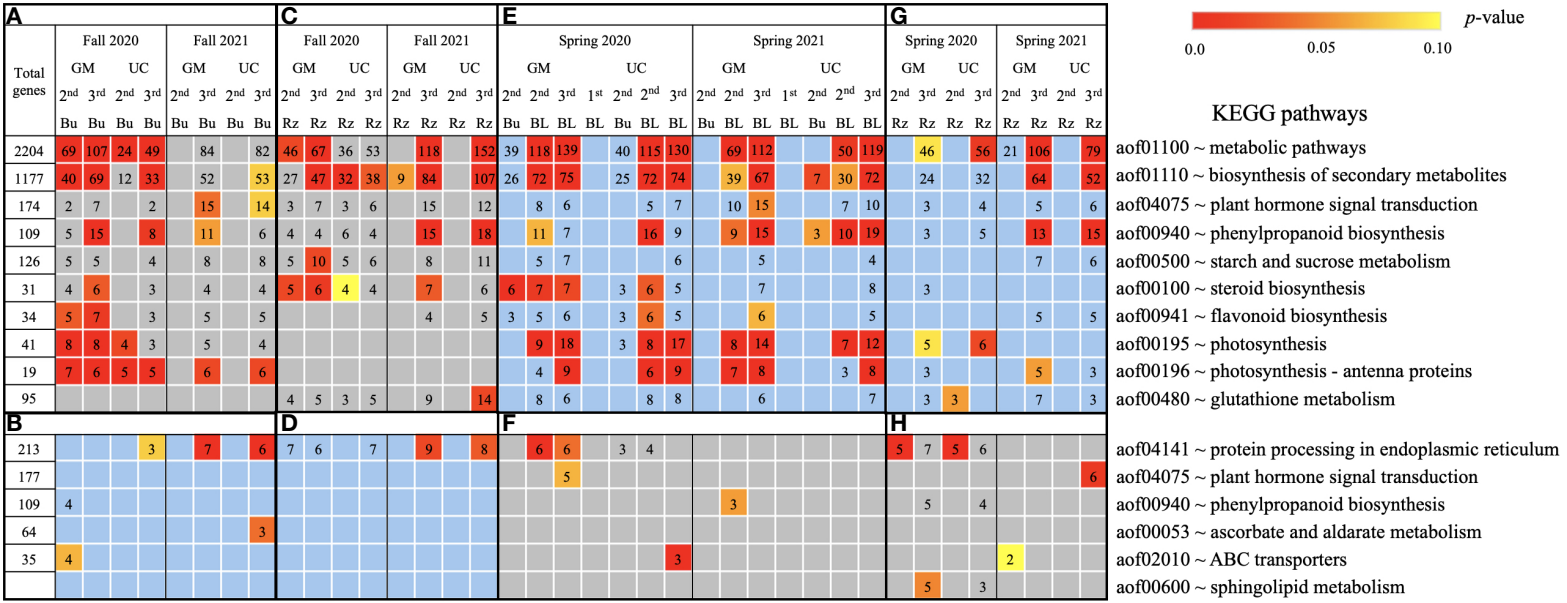
Metabolic pathways using KEGG database assigned to genes differentially expressed relative to the first harvest for asparagus cultivars ‘Guelph Millennium’ (GM) and ‘UC157’ (UC). GO terms **(A)** downregulated and **(B)** upregulated in dormant buds (Bu) during the fall (2020 and 2021); GO terms **(C)** downregulated and **(D)** upregulated in rhizomes (Rz) during the fall (2020 and 2021); GO terms **(E)** upregulated and **(F)** downregulated in dormant and growing buds (BL) during the spring (2020 and 2021); GO terms **(G)** upregulated and **(H)** downregulated in rhizomes during the spring (2020 and 2021). The number of genes assigned to each GO term are presented in the cells; blank boxes equal zero. Within upregulated (blue area) and downregulated (grey area) genes, significantly enriched terms are color-coded based on an adjusted *p*-value i.e., false discovery rate of less than 0.1 (red most significant). 1^st^ harvest; 2^nd^ harvest; 3^rd^ harvest.

Based on GO term and metabolic pathway analysis, genes downregulated in the fall of 2020 and 2021 were found to be associated with physiological components and processes such as carbohydrates, oxidative stress, cell wall, membranes, photosynthesis, hormones, steroids, flavonoids, and glutathione metabolism (**Figures 4**–**6A, C**). The number of genes assigned to each term/pathway was consistent with the LT_50_ patterns; more genes were assigned to GM than UC at the second and third harvests of 2020 and/or similar numbers of genes were assigned to both cultivars at the third harvest in 2021 (**Supplementary Table 11**). Genes involved in these terms and pathways were downregulated in the dormant buds and rhizomes of both cultivars which suggests that the same processes are affected in both tissues except for photosynthesis and glutathione-related terms/pathways which were downregulated only in the dormant buds and rhizomes, respectively. Additionally, genes involved in cell cycle/division, ethylene signaling, trehalose biosynthesis, carbon metabolism, and biosynthesis of amino acids were also downregulated in the dormant buds and/or rhizomes of both cultivars during the fall of both years, but the number of genes assigned to each term/pathway was not consistent with the LT_50_ patterns (**Supplementary Figure 3**; **Supplementary Table 11**).

In the fall of 2020 and 2021, genes upregulated in the dormant buds and/or rhizomes of both cultivars were assigned to GO terms and pathways that included carbohydrates, circadian rhythm, response to heat/water, endoplasmic reticulum, membranes, and ABC transporters (**Figures 4**–**6B, D**). However, the numbers of genes assigned to upregulated GO terms/metabolic pathways were not consistent with the LT_50_ patterns, differences or similarities, between the cultivars except for carbohydrates in dormant buds (**Supplementary Table 11**).

During the spring of 2020 and 2021, an opposite pattern of expression was observed compared to fall. GO terms and pathways downregulated in the fall were upregulated in the growing buds and rhizomes of both cultivars (**Figures 4**–**6E, G**). For most GO terms and pathways upregulated in spring, the number of upregulated genes assigned to each pathway was highest and similar between cultivars at the third harvest relative to the first harvest, consistent with the LT_50_ results (**Supplementary Table 11**). Similarly, GO terms and pathways upregulated in the fall were assigned to downregulated genes in the spring, but did not exhibit specific patterns that could be related to LT_50_ (**Figures 4**–**6F, H**; **Supplementary Table 11**).

### 3.6 Identification of DE genes involved in fall acclimation and spring deacclimation

Many carbohydrate genes were DE in both buds and rhizomes of GM and UC during fall acclimation and spring deacclimation. During the fall of both years, genes involved in fructose and mannose metabolism such as *FRUCTOSE-BISPHOSPHATE ALDOLASE (FBA)* and *BETA-FRUCTOFURANOSIDASE 3*, and genes involved in starch and sucrose metabolism such as *BETA-GLUCOSIDASE BOGH3B* and *ENDOGLUCANASE 3* were downregulated in the dormant buds and rhizomes of both cultivars (**Table 3**; **Supplementary Figure 4A**). On the other hand, genes involved in galactose metabolism such as *GALACTINOL-SUCROSE GALACTOSYLTRANSFERAS (RFS1)* and *GALACTINOL SYNTHASE 1 (GOLS1)*, and genes involved in starch and sucrose metabolism such as *SUCROSE-PHOSPHATE SYNTHASE 2 (SPS2), TREHALOSE-PHOSPHATE PHOSPHATASE F (TPPF)*, and *SUCROSE SYNTHASE 2 (SS2)* were upregulated in the dormant buds and/or rhizomes of both cultivars in fall 2020 and 2021 (**Table 3**; **Supplementary Figure 4A**). Additionally, two bidirectional sugar transporter (SWEET) genes (*SWEET13, SWEET14*) were downregulated, and two *SWEET1a* genes were upregulated in the dormant buds and rhizomes of both cultivars during fall (**Table 3**; **Supplementary Figure 4B**). All carbohydrate genes were down or upregulated (either in dormant buds or rhizomes) to higher levels in GM as compared to UC at the second and third harvests in 2020 and to similar levels in both cultivars in 2021 which was consistent with the LT_50_ differences or similarities between cultivars.

Other downregulated genes that exhibited expression patterns similar to LT_50_ in the dormant buds and/or rhizomes of both cultivars during fall of 2020 and 2021 (**Table 3**) included plant hormone genes such as auxin binding/induced proteins, ethylene-responsive transcription factors, xyloglucan endotransglucosylase/hydrolase (XTH) proteins, and gibberellin (GA) 20-oxidases (**Supplementary Figure 5**), chlorophyll a-b binding proteins and photosynthesis genes (**Supplementary Figure 6**), proline degradation and transport genes (**Supplementary Figure 7A**), peroxidases (**Supplementary Figure 8**), steroid and flavonoid biosynthesis genes (**Supplementary Figures 9A, B**), and glutathione transferases (GST) (**Supplementary Figure 9C**). Transcripts for genes such as GA-regulated proteins (**Supplementary Figure 5**), proline synthesis genes (**Supplementary Figure 7**), various heat shock proteins (HSP) (**Supplementary Figure 10A**), and late embryogenesis abundant (LEA) proteins (**Supplementary Figure 10C**) were induced to high levels (consistent with LT_50_ patterns) in the dormant buds and rhizomes of both cultivars in the fall of both years (**Table 3**). Genes involved in circadian rhythm were also upregulated in both tissues and cultivars during fall 2020, although levels of expression were similar between cultivars at the second and third harvests of 2020 (**Supplementary Figure 10B**). During spring, the above genes showed the opposite expression pattern in the growing buds and/or rhizomes of both cultivars either in 2020 or in 2021 (**Table 3**).

### 3.7 Transcription factors encoding DE genes

Seventeen TF families were identified among the genes DE during fall and/or spring (**Supplementary Figure 11**). Of these, NAC was predominant followed by MYB, bHLH, WRKY and AP2/ERF families (**Supplementary Figure 11**). Members of the MYB, bHLH, WRKY, and AP2/ERF families, previously identified in the plant cold stress response pathway, were downregulated during fall and upregulated during spring (**Supplementary Figure 12**). Specifically, five WRKY (**Supplementary Figure 12A**), 10 MYB (**Supplementary Figure 12B**), seven bHLH (**Supplementary Figure 12C**), and eight AP2/ERF (**Supplementary Figure 12D**) TFs followed these seasonal expression patterns during falls and springs of both years. However, two AP2/ERF genes (*DEHYDRATION-RESPONSIVE ELEMENT-BIND PROTEIN 2C* and *AP2-LIKE ETHYLENE-RESPONSIVE TRANSCRIPTION FACTOR BBM)* (**Supplementary Figure 12D**) and one MYB genes (*MYB-RELATED PROTEIN ZM1*) (**Supplementary Figure 12B**) were upregulated during fall and downregulated during spring. All identified TFs showed expression patterns that could be related to LT_50_ differences or similarities among cultivars in the fall or spring of both years.

### 3.8 Validation of RNA-Seq results

qRT-PCR analysis indicated that during the fall of 2020 and 2021, three genes (*FBA, CHL6, PRODH2*) were downregulated and four genes (*RFS1, CCA1, LEA14A, SS2*) were upregulated in the dormant buds (**Supplementary Figures 13A, B**) and rhizomes (**Supplementary Figures 14A, B**) of both cultivars. The expression patterns of all seven genes were consistent with the RNA-Seq data. In the spring of 2020 and 2021, these genes showed opposite expression; *FBA, CHL6* and *PRODH2* genes were upregulated and *RFS1, CCA1, LEA14A* and *SS2* genes were downregulated in the dormant and growing buds (**Supplementary Figures 13C, D**) and rhizomes (**Supplementary Figures 14C, D**) of both cultivars. The degree of upregulation or downregulation of seven genes in buds as well as in rhizomes during spring was highest at the third harvest, which was also consistent with RNA-Seq observations. Linear regression analysis of log_2_fold changes obtained by RNA-Seq and relative expression obtained by qRT-PCR showed high positive correlations (R^2^ values ranging from 0.70 to 0.89) between the gene expression assessed by the two approaches (**Supplementary Figure 15**). Taken together, these results confirmed the reliability of the RNA-Seq data.

## 4 Discussion

Asparagus cultivars GM and UC acclimated in the fall and deacclimated in the spring, showing different or similar levels of freezing tolerance related to harvest date and year. The number of DE genes increased in both cultivars during acclimation and deacclimation, with majority of genes being downregulated and upregulated during fall and spring, respectfully. Gene expression patterns in the dormant buds during fall 2020, dormant buds and rhizomes during fall 2021, and growing buds and rhizomes during spring 2021 were consistent with the freezing tolerance (LT_50_) differences or similarities between the cultivars. DE genes were assigned to several GO terms/pathways such as “carbohydrate metabolic pathway”, “plant hormone signal transduction”, “response to oxidative stress”, “proline metabolism”, “lipid metabolic process”, “microtubule”, “circadian rhythm”, “protein processing in endoplasmic reticulum.” In addition to these known processes associated with freezing tolerance, photosynthesis and cell wall/membrane-related GO terms/pathways were identified. Specifically, genes such as *FBA, GOLS1, RFS1, TPPF, P5CS, PRODH2*, *LEA, HSP*, *CCA1*, and *LNK* could be involved in asparagus freezing tolerance. Individual genes involved in photosynthesis, hormone signal transduction and cellular detoxification were also related to freezing tolerance levels.

During fall, the freezing tolerance of GM and UC increased as the soil temperatures decreased (**Table 2**). In 2020, GM showed early acclimation (high freezing tolerance) as compared to UC and maintained a high freezing tolerance throughout the sampling period. In 2021, both GM and UC displayed no change in their LT_50_ values in early to mid-fall. However, both cultivars achieved the same levels of freezing tolerance by late fall. The observed differences in freezing tolerance patterns between the two years could be explained by soil temperature variations. In 2020, soil temperature exhibited a gradual decline from the first harvest to the third, contributing to the increase in freezing tolerance and observed differences between the two cultivars. In 2021, soil temperatures only decreased by the third harvest. Moreover, the third harvest of 2021 was delayed by one week in comparison to the third harvest of 2020, which could have provided both cultivars sufficient time to acclimate completely and achieve the same levels of freezing tolerance. Panjtandoust and Wolyn (2016a) reported that GM had higher freezing tolerance compared to UC during early October, and both cultivars had the same levels of freezing tolerance by late fall (mid-November).

During spring 2021, the freezing tolerance of cultivars decreased as plants deacclimated with the increase in soil temperatures (**Table 2**), and UC lost its freezing tolerance earlier in the season as compared to GM. These results are consistent with those of Panjtandoust and Wolyn (2016b). LT_50_ did not change for the cultivars over harvest dates in 2020, despite observable differences in bud growth. The spring 2020 LT_50_ data may be less precise than those of 2021 due to the number of temperatures used to estimate the parameter, one less than in 2021.

Consistent with changing soil temperatures and levels of freezing tolerance in the fall, the cultivars showed distinct gene expression patterns. In fall 2020, more genes were DE in the dormant buds of GM than UC at the second and third harvests when GM exhibited higher freezing tolerance than UC. However, a similar number of genes were DE in the rhizomes of both cultivars at both harvests despite the differences in freezing tolerance (**Table 2**). Consequently, genes DE in dormant buds may be most important for asparagus freezing tolerance. The majority of DE genes were downregulated in both cultivars in the fall (**Figure 3**), indicating an attenuation of most cellular processes as the cultivars acclimated to cold and became dormant. Functional analysis revealed that those downregulated were involved in cell division, biosynthesis of secondary metabolites, plant hormone signal transduction, and photosynthesis. The downregulation of genes and cellular processes during fall acclimation has also been reported in *Arabidopsis*, alfalfa, tea *(Camellia sinensis* L.), and evergreen shrubs (*Rhododendron anthopogon* D.) (Wang et al., 2013; Byun et al., 2014; Rathore et al., 2022). Upregulation of asparagus genes related to carbohydrate metabolism, circadian rhythm, heat shock proteins, and LEA proteins during fall cold acclimation suggests these genes and pathways may be critical positive regulators of freezing tolerance.

In the spring, gene expression increased in the growing buds and rhizomes of both cultivars as plants deacclimated, freezing tolerance decreased (**Table 2**) and buds started growing. Spring deacclimation involves reversing some of the adaptations made during fall acclimation, allowing the plants to resume normal physiological activities. Genes associated with photosynthesis, cell division and other growth-related processes and pathways which were downregulated during fall acclimation were upregulated during spring deacclimation. Similarly, genes that were upregulated during fall acclimation in response to cold were downregulated in spring. Genes showing expression patterns consistent with the increase and decrease of freezing tolerance in fall and spring, respectively, validates their importance for further study.

### 4.1 Carbohydrate metabolism

“Carbohydrate metabolic process” was one of the most enriched and abundant GO terms among DE genes during fall and spring (**Figure 4**). Several other studies have revealed that the carbohydrate metabolism pathway is most sensitive under cold stress (Xu et al., 2017; Yang et al., 2019; Dong et al., 2020; Aguayo et al., 2023). Carbohydrates, particularly soluble sugars, play multifaceted roles in the development of freezing tolerance, acting as cryoprotectants, contributing to osmotic adjustment, stabilizing cell membranes, and providing energy for recovery after cold stress (Fürtauer et al., 2019). Generally, the concentrations of soluble sugars increase in the roots of woody plants during fall acclimation, indicating the mobilization of storage carbohydrates to achieve maximum freezing tolerance (Ambroise et al., 2020). Conversely, during spring deacclimation, soluble sugar concentrations decrease to support bud growth (Trischuk et al., 2014; Shin et al., 2015). Previous studies in asparagus have reported an increase in raffinose concentrations in rhizomes of GM and UC until mid-October but a decrease late in the season for both cultivars (Landry and Wolyn, 2011). Raffinose concentrations also decreased in the rhizomes of both cultivars during spring deacclimation (Panjtandoust and Wolyn, 2016b). Moreover, accumulation of raffinose and its correlation to increased freezing tolerance has also been reported in *Arabidopsis*, sugar beet (*Beta vulgaris* L.), maize (*Zea mays* L.*)*, and alfalfa (Han et al., 2019; Keller et al., 2021). In the current study, two *RFS1* and one *GOLS1* genes (enzymes involved in the synthesis of raffinose) were highly upregulated in the fall (consistent with freezing tolerance differences) in the dormant buds and rhizomes of both cultivars (**Table 3**; **Supplementary Figure 4A**). In spring, these genes were downregulated as freezing tolerance decreased. Increased transcription levels of the *GOLS1* and *RFS1* genes as well as the accumulation of raffinose in response to cold stress have been reported in *Arabidopsis*, rice seedlings, and grapes (Saito and Yoshida, 2011; Noronha et al., 2022).

The *FBA* gene was downregulated in the dormant buds of both cultivars in the fall and highly upregulated in the spring (**Table 3**; **Supplementary Figure 4**). *FBA* catalyzes the reversible conversion of fructose-1,6 bisphosphate into dihydroxyacetone phosphate and glyceraldehyde 3-phosphate during glycolysis (**Supplementary Figure 4C**), thereby generating metabolites for starch biosynthesis (Zeng et al., 2014). In tomato (*Solanum lycopersicum* L*.)*, chilling stress decreased activity of *FBA7* which eventually led to a decrease in net photosynthetic rate, ribulose-1,5-bisphosphate, soluble sugar and sucrose content, stem diameter, dry weight and seed size (Cai et al., 2018). *FBA4* overexpression in tomato transgenic lines increased the expression and activities of other main enzymes in the Calvin cycle, net photosynthetic rate, seed size and stem diameter and seed germination tolerance under cold stress (Cai et al., 2022). These data suggest that downregulation of *FBA* in asparagus during fall could be involved in decreasing growth and soluble sugar content, especially for sucrose, eventually leading to dormancy and increased freezing tolerance. Upregulation of *FBA* in the spring could be involved in active bud growth.

Two *β-FRUCTOFURANOSIDASEs* were upregulated at the second harvest of fall 2020 in the dormant buds of GM only but were downregulated in rhizomes and dormant buds of both cultivars at the third harvest (**Table 3**; **Supplementary Figure 4A**). *β-FRUCTOFURANOSIDASE* hydrolyzes sucrose to produce glucose and fructose (**Supplementary Figure 4C**), and also releases fructose from fructans, thereafter playing an important role in osmoprotection and energy production in plants under low temperature stress (Khan et al., 2017). Additionally, an *SS2* gene was upregulated in the dormant buds and rhizomes of both asparagus cultivars as plants acclimated and increased freezing tolerance in the fall, but was downregulated during spring (**Table 3**; **Supplementary Figure 4A**). The *SS* gene, particularly in sink tissues, can cleave sucrose into fructose and either UDP-glucose or ADP-glucose (**Supplementary Figure 4C**) which could act as osmoprotectants during cold stress (Stein and Granot, 2019). The *TPPF* gene, involved in the synthesis of trehalose which plays an important role in stabilizing membranes and proteins at low temperatures (Zang et al., 2011), was also upregulated in dormant buds and rhizomes of both cultivars during fall acclimation (**Table 3**; **Supplementary Figure 4A**). *A. thaliana* plants subjected to chilling stress also showed an increased in trehalose and transgenic plants overexpressing *TPPF* accumulated trehalose and displayed a significant increase in freezing tolerance (Iordachescu and Imai, 2008). Overall, carbohydrate genes upregulated in asparagus during fall acclimation were found to be involved in the production of raffinose, fructose, glucose, and trehalose. All these sugars are known to be involved in freezing tolerance and act as cryoprotectants in many plant species.

### 4.2 Plant hormone signal transduction

Many genes involved in plant hormone signal transduction showed differential expression either in the buds or rhizomes during fall acclimation and spring deacclimation (**Figure 6**; **Table 3**). Hormones act as signaling molecules that play key roles in regulating gene expression under cold stress. Growth-promoting hormones, such as auxin and gibberellin regulate plant growth and development, including cell elongation and division under optimum conditions (Eremina et al., 2016). Under cold stress, a decline in endogenous auxin levels has been documented in leaves and roots of *A. thaliana* (Sharma et al., 2015). Cold stress also inhibited root growth, meristem size and cell number, repressing the division potential of meristematic cells by decreasing auxin accumulation, possibly because cold stress reduced the expression of auxin transport and biosynthesis-related genes (Zhu et al., 2015). GA 20-oxidase is a regulatory enzyme for the synthesis of biologically active GA in plants. The expression of GA 20-oxidase decreased in the roots and leaves of Zoysiagrass (*Zoysia japonica* L.) under low temperatures (Dai et al., 2012). In asparagus, the downregulation of genes involved in auxin and GA biosynthesis, such auxin binding proteins and GA 20-oxidases, in response to low temperatures during fall could be linked to induction of dormancy and acclimation especially in the buds. Conversely, the upregulation of these genes during spring could be associated with active growth of buds and a decrease in freezing tolerance (**Supplementary Figure 5**).

Four *XTH* genes were downregulated in dormant buds of GM and UC during the fall (**Supplementary Figure 5**). Xyloglucan is a soluble hemicellulose in the primary cell wall of plants. *XTH* is involved in the modification of cell wall structure by cleaving and rejoining xyloglucan molecules. The downregulation of XTH genes can lead to alterations in cell wall composition such as changes in xyloglucan content which can strengthen the cell wall and provide improved protection against freezing-induced damage (Hayashi and Kaida, 2011; Cheng et al., 2021). Similarly, downregulation of *XTH* genes in asparagus and their expression, consistent with freezing tolerance differences between cultivars, could play a potential role in cell wall remodeling for increased freezing tolerance.

### 4.3 Photosynthesis

Many photosynthesis genes, including those encoding chlorophyll a-b binding proteins, and photosystem I and photosystem II proteins were downregulated only in the dormant buds of asparagus cultivars during fall 2020 and 2021 (**Figures 4**–**6**). Although asparagus buds are not active sites of photosynthesis in the fall, gene expression patterns were consistent with observed differences or similarities in freezing tolerance between cultivars (**Supplementary Figure 6**). In spring, when these dormant buds emerge as spears and become active sites of photosynthesis, photosynthesis genes were highly upregulated (**Supplementary Figure 6**). Decreases in temperature result in cessation of growth which greatly reduces carbon sink capacity and subsequently slows cellular respiration and induces a negative feedback regulation of carbon assimilation (Banerjee and Roychoudhury, 2019). To compensate for a reduced energy sink, plants lower their capacity for harvesting sunlight by adjusting photosynthetic pigments and by downregulating the expression of photosynthesis genes (Rapacz et al., 2008). In asparagus, downregulation of photosynthesis-related genes during fall could be associated with dormancy and cessation of bud growth. In spring, the buds could be sensing the increase in temperature, signifying imminent growth and light exposure, prompting an increase in the expression of photosynthesis genes to prepare buds and ultimately spears and fern for photosynthesis.

### 4.4 Proline metabolism

In addition to carbohydrate genes, two *P5CS* genes were upregulated and one *PRODH2* gene was downregulated in the dormant buds and rhizomes of GM and UC during fall (**Table 3**; **Supplementary Figure 7A**). *P5CS* genes are involved in proline synthesis in the cytosol and catalyze the production of proline from glutamate via the intermediate delta-1- pyrroline-5-carboxylate (**Supplementary Figure 7B**) (Verslues and Sharma, 2010). *PRODH* catalyzes the oxidation of proline to -1-pyrroline-5-carboxylate which is converted into glutamate during proline catabolism in mitochondria (**Supplementary Figure 7B**) (Verslues and Sharma, 2010). Previously, proline concentrations increased in asparagus rhizomes as temperature decreased in the fall, with GM having higher concentrations than UC (Landry and Wolyn, 2011; Kim and Wolyn, 2015; Panjtandoust and Wolyn, 2016a). Proline has also been known to act as a compatible solute and play a significant role to prevent cellular dehydration by increasing the osmotic potential during cold stress. The accumulation of proline in cells during cold acclimation helps to maintain membrane integrity, stabilize proteins, and protect cells from oxidative stress (Zulfiqar and Ashraf, 2022). In *A. thaliana*, a more than 2-fold increase in proline occurred after a 4 h exposure to cold stress and was followed by a continuous and dramatic increase up to 130-fold after 96 h (Kaplan et al., 2007). Thus, upregulation of *P5CS* genes and downregulation of *PRODH2* could be leading to high concentrations of proline in asparagus cultivars in the fall. Moreover, *P5CS* genes were upregulated to a higher extent in buds of GM than UC at the second harvest of fall 2020 which coincided with the higher freezing tolerance of GM than UC and suggested a role for proline in the acquisition of freezing tolerance.

Additionally, a *PROLINE TRANSPORTER 2* was also downregulated in dormant buds and rhizomes during fall (**Table 3**; **Supplementary Figure 7A**), potentially reducing transport of proline from cytosol to mitochondria and increasing cytoplasmic concentrations. During spring deacclimation, these proline biosynthesis pathway genes exhibited an opposite pattern of expression; *P5CS* genes were downregulated and *PRODH2* and *PROLINE TRANSPORTER 2* genes were upregulated in growing buds and rhizomes of asparagus cultivars, consistent with a previous study which showed levels of proline decreased in rhizomes of GM and UC during spring deacclimation (Panjtandoust and Wolyn, 2016b).

### 4.5 Response to oxidative stress and cellular detoxification

Many genes involved in the biosynthesis of secondary metabolites (such as phenylpropanoids, steroids, and flavonoids) and glutathione metabolism were also downregulated in asparagus during fall and upregulated during spring (**Figure 6**). Phenylpropanoid biosynthesis genes were also assigned to “response to oxidative stress” and “hydrogen peroxide catabolic process” (**Figure 4**). In plants, ROS are produced in excess under cold stress, causing oxidative damage, usually associated with the peroxidation of membrane lipids in which peroxidase enzymes play important roles (Choudhury et al., 2017; Chen et al., 2022). Downregulation of peroxidase genes during fall acclimation in asparagus (**Supplementary Figure 8**) could prevent oxidative damage caused by over accumulation of ROS under cold stress. Plant antioxidant systems (such as those utilizing steroids, flavonoids, and glutathione) could also protect plants against oxidative stress by detoxification of ROS (Song et al., 2021). GSTs are part of the glutathione-ascorbate cycle, an important cellular antioxidant system that helps regulate the balance between reduced and oxidized forms of glutathione (Song et al., 2021). The downregulation of steroid and flavonoid biosynthesis genes and GSTs (**Supplementary Figure 9**) could be involved in reprogramming plant metabolism to prioritize other stress response mechanisms such as the accumulation of osmoprotectants and secondary metabolites to help plants cope with cold stress.

### 4.6 Involvement of late embryogenesis abundant protein and heat shock protein genes

During fall acclimation, asparagus cultivars showed upregulation of genes encoding protective proteins such as LEA and HSP in dormant buds and rhizomes (**Table 3**; **Supplementary Figure 10**). An increase in LEA as well as in HSP gene expression has also been associated with low temperature acclimation in several species such as maize, evergreen shrubs and peach (*Prunus persica* L*.)* (Shin et al., 2015; Elkelish et al., 2020; Rathore et al., 2022). LEA proteins, also referred to as hydrophilins, accumulate in plants in response to cold stress and play important roles such as cryoprotectants, and membrane and protein stabilizers (Banerjee and Roychoudhury, 2016). HSPs have also been known to contribute to cellular homeostasis in plants. The upregulation of HSPs in response to cold stress could protect proteins from misfolding or facilitate the degradation of misfolded and damaged proteins to maintain cellular homeostasis (Bourgine and Guihur, 2021). The increased transcript levels of LEA protein and HSP genes during asparagus acclimation, consistent with freezing tolerance levels of cultivars, and downregulation of LEA/HSP genes during deacclimation, along with a decrease in freezing tolerance, indicates a potential role for these genes to protect plants from damaging freezing stress.

### 4.7 Circadian rhythm regulation

Many circadian rhythm genes *(CCA1, LNK1, LNK2*, *PRR95)* were upregulated in the dormant buds and rhizomes of both cultivars during fall acclimation in 2020 (**Table 3**; **Supplementary Figure 10B**). Circadian rhythm genes induced the expression of many cold regulated genes and pathways in various perennials such as poplar, Eucalyptus, and chestnut (*Castanea dentata* L.) (Johansson et al., 2015). *Populus* trees with increased expression of *LHY1* and *LHY2* showed increased *CBF1* expression in response to cold and had increased freezing tolerance (Ibáñez et al., 2010). Conversely, the loss of *LHY1* and *LHY2* expression led to loss of *CBF1* expression and reduced freezing tolerance (Ibáñez et al., 2010). In asparagus, circadian rhythm genes were upregulated to similar levels in dormant buds and rhizomes of both cultivars at the second and third harvests of fall 2020 (**Table 3**, **Supplementary Figure 10B**) although GM showed higher freezing tolerance than UC at both harvests of fall 2020 (**Figure 1B**). Upregulation of circadian rhythm genes could be involved in the induction of downstream genes associated with the cold signaling pathways in asparagus, eventually leading to the observed freezing tolerance differences between cultivars.

### 5 Conclusion

Overall, results indicated that genes involved in carbohydrate metabolic process (*RFS1, GOLS1, FBA, TPPF*), proline metabolism (*P5CS* and *PRODH2*), auxin and GA biosynthesis (auxin binding proteins and GA 20-oxidases), photosynthesis, LEA proteins, HSPs, circadian rhythm (*CCA1, LNK1, LNK2*), and biosynthesis of secondary metabolites may regulate varying levels of freezing tolerance observed in asparagus cultivars GM and UC and may be considered candidates for further investigation and plant improvement. Candidate genes, after functional verification, can be used in marker-assisted selection. Breeders can select plants with desired gene variants through DNA testing, allowing for improved precision and efficiency in breeding. Modifying expression of specific candidate genes through genetic engineering may also lead to enhanced freezing tolerance.

## Data Availability

The datasets presented in this study can be found in online repositories. The names of the repository/repositories and accession number(s) can be found in the article/**Supplementary Material**.
