# Supplementary material for: Transcriptomic analysis of Asparagus officinalis cultivars with varying levels of freezing tolerance over fall acclimation and spring deacclimation periods
Source: Front Plant Sci. 2024 Aug 16;15:1442784. doi: 10.3389/fpls.2024.1442784 (PMC11361922; doi:10.3389/fpls.2024.1442784)
Supplement: Supplementary file 1 [file DataSheet1.docx]

Supplementary Material

# Transcriptomic analysis of *Asparagus officinalis* cultivars with varying levels of freezing tolerance over fall acclimation and spring deacclimation periods

Arshdeep Singh Gill^1^, David J. Wolyn^1*^

^1^Department of Plant Agriculture, University of Guelph, Guelph, Ontario, Canada, N1G 2W1

*** Correspondence:**David J. Wolyn
dwolyn@uoguelph.ca

# Supplementary Tables

Supplementary Table 1 Asparagus crown harvest dates during fall and spring seasons in 2020 and 2021.

| **Planting** | **Harvest in Fall** | **Harvest in Spring** |
| --- | --- | --- |
| 24 June 2019 | 22 September 2020 | 09 March 2020 |
|  | 19 October 2020 | 25 March 2020 |
|  | 05 November 2020 | 06 April 2020 |
| 16 June 2020 | 21 September 2021 | 22 March 2021 |
|  | 13 October 2021 | 29 March 2021 |
|  | 10 November 2021 | 14 April 2021 |

Supplementary Table 2 Primers used in the quantitative reverse transcription (qRT)-PCR for the validation of RNA-Seq results in asparagus samples.

| **Gene ID** | **Gene annotation** |  | **Forward (F) and reverse (R) primers (5’ to 3’)** |
| --- | --- | --- | --- |
| A4U43_C09F1540 | *ACTIN* | F  R | CCAAGGCAGAGTACGATGAA  CCACCTCAAGACAGCTAGATAC |
| A4U43_C02F7310 | *FRUCTOSE-BISPHOSPHATE ALDOLASE*, chloroplastic (*FBA*) | F  R | GATACTGTTGGACGGTGAG  GGAGCTTGAGGGTGTATTC |
| A4U43_C05F21680 | *GALACTINOL-SUCROSE GALACTOSYLTRANSFERASE 1* (*RFS1*) | F  R | CCACCCTATGGCTGAATAC  CACGAGCTGGATCTGAAA |
| A4U43_C07F20660 | *CHLOROPHYLL A-B BINDING PROTEIN 6*, chloroplastic (*CHL6*) | F  R | GGTTTCGATCCTCTTCGG  GAGCCTGTACCCAGTTTC |
| A4U43_C10F16170 | *PROLINE DEHYDROGENASE 2*, mitochondrial (*PRODH2*) | F  R | CAGAGGAGCTCGGAATATC  GAAGGAGGTAAGGGATGAC |
| A4U43_C01F15360 | protein *CCA1* | F  R | CACAGGAAGAGGGAGTAGA  GCTACCTTGCCCAGTAATAG |
| A4U43_C01F9930 | *LATE EMBRYOGENESIS ABUNDANT PROTEIN 14-A* (*LEA14A*) | F  R | GAGGTCTCCGTTAACAATCC  GAACCTCCAGCTTCGTTAC |
| A4U43_C07F25270 | *SUCROSE SYNTHASE 2* (*SS2*) | F  R | GACATGTCCATCTACTTCCC  GGGCTTGTTCCTATCCTTC |

**Supplementary Table 3** Overview of sequencing and mapping of fall 2020 RNA-Seq samples in asparagus.

| **Sample** | **Biological replicate** | **Raw reads** | **Reads percent with Q score >30** | **Uniquely mapped reads (percent)** | **Multimapped reads (percent)** | **Unmapped reads (percent)** |
| --- | --- | --- | --- | --- | --- | --- |
| F20.1^st^.GM.Bu | Rep 1 | 21,701,297 | 96.1 | 18,938,068 (87.3) | 1,270,937 (5.8) | 1,492,292 (6.9) |
|  | Rep 2 | 20,249,569 | 94.9 | 17,291,734 (85.4) | 1,596,389 (7.9) | 1,361,446 (6.7) |
|  | Rep 3 | 20,604,682 | 95.8 | 17,782,378 (86.3) | 1,269,205 (6.2) | 1,553,099 (7.5) |
| F20.1^st^.GM.Rz | Rep 1 | 25,204,091 | 94.9 | 21,874,691 (86.8) | 1,497,476 (5.9) | 1,831,924 (7.3) |
|  | Rep 2 | 24,115,391 | 96.8 | 20,662,439 (85.7) | 2,039,305 (8.5) | 1,413,647 (5.9) |
|  | Rep 3 | 26,727,965 | 95.1 | 23,174,073 (86.7) | 1,448,167 (5.4) | 2,105,725 (7.9) |
| F20.2^nd^.GM.Bu | Rep 1 | 22,186,614 | 95.7 | 19,306,454 (87) | 1,517,684 (6.8) | 1,362,476 (6.1) |
|  | Rep 2 | 26,029,006 | 95.9 | 22,165,664 (85.2) | 2,344,226 (9) | 1,519,116 (5.8) |
|  | Rep 3 | 25,409,491 | 94.5 | 21,739,860 (85.6) | 1,527,631 (6) | 2,142,000 (8.4) |
| F20.2^nd^.GM.Rz | Rep 1 | 20,819,539 | 95 | 17,894,170 (86) | 1,379,729 (6.6) | 1,545,640 (7.4) |
|  | Rep 2 | 23,584,610 | 95.5 | 20,453,156 (86.7) | 1,500,683 (6.4) | 1,630,771 (6.9) |
|  | Rep 3 | 15,286,611 | 95.6 | 13,141,523 (86) | 1,047,238 (6.8) | 1,097,850 (7.2) |
| F20.3^rd^.GM.Bu | Rep 1 | 22,626,724 | 95.9 | 19,420,777 (85.8) | 1,806,241 (8) | 1,399,706 (6.3) |
|  | Rep 2 | 18,937,852 | 94.7 | 16,321,409 (86.2) | 1,321,892 (7) | 1,294,551 (6.8) |
|  | Rep 3 | 22,255,291 | 95.6 | 19,030,168 (85.5) | 1,860,053 (8.4) | 1,365,070 (6.1) |
| F20.3^rd^.GM.Rz | Rep 1 | 26,944,941 | 94 | 22,919,092 (85.1) | 1,862,009 (6.9) | 2,163,840 (8) |
|  | Rep 2 | 24,138,135 | 96.6 | 20,834,396 (86.3) | 1,396,494 (5.8) | 1,907,245 (7.9) |
|  | Rep 3 | 25,199,802 | 95.2 | 21,505,605 (85.3) | 1,856,868 (7.4) | 1,837,329 (7.3) |
| F20.1^st^.UC.Bu | Rep 1 | 23,935,906 | 95.4 | 20,325,911 (84.9) | 2,131,984 (8.9) | 1,478,011 (6.2) |
|  | Rep 2 | 25,062,247 | 95.9 | 21,071,062 (84.1) | 2,328,784 (9.3) | 1,662,401 (6.6) |
|  | Rep 3 | 23,572,449 | 95.8 | 20,096,295 (85.3) | 1,869,435 (7.9) | 1,606,719 (6.8) |
| F20.1^st^.UC.Rz | Rep 1 | 25,031,015 | 97.2 | 21,727,317 (86.8) | 1,817,172 (7.3) | 1,486,526 (5.9) |
|  | Rep 2 | 20,634,266 | 96.4 | 17,580,837 (85.2) | 1,756,357 (8.5) | 1,297,072 (6.3) |
|  | Rep 3 | 22,475,957 | 96.6 | 19,794,966 (88.1) | 1,190,274 (5.3) | 1,490,717 (6.6) |
| F20.2^nd^.UC.Bu | Rep 1 | 20,037,018 | 95.9 | 17,381,925 (86.7) | 1,417,127 (7.1) | 1,237,966 (6.2) |
|  | Rep 2 | 26,060,287 | 94.5 | 22,477,959 (86.3) | 1,798,154 (6.9) | 1,784,174 (6.8) |
|  | Rep 3 | 59,066,536 | 99 | 49,963,047 (84.6) | 4,518,626 (7.6) | 4,584,863 (7.8) |
| F20.2^nd^.UC.Rz | Rep 1 | 25,540,856 | 94.9 | 21,752,968 (85.2) | 1,948,738 (7.6) | 1,839,150 (7.2) |
|  | Rep 2 | 27,041,977 | 96.4 | 23,204,422 (85.8) | 2,018,063 (7.5) | 1,819,492 (6.7) |
|  | Rep 3 | 19,523,235 | 93.9 | 15,746,623 (80.7) | 1,480,409 (7.5) | 2,296,203 (11.8) |
| **Sample** | **Biological replicate** | **Raw reads** | **Reads percent with Q score >30** | **Uniquely mapped reads (percent)** | **Multimapped reads (percent)** | **Unmapped reads (percent)** |
| F20.3^rd^.UC.Bu | Rep 1 | 26,976,958 | 94.8 | 22,626,077 (83.9) | 2,561,774 (9.5) | 1,789,107 (6.6) |
|  | Rep 2 | 24,740,408 | 95.6 | 21,132,841 (85.4) | 2,021,213 (8.2) | 1,586,354 (6.4) |
|  | Rep 3 | 26,198,628 | 94.8 | 22,101,262 (84.4) | 2,572,335 (9.8) | 1,525,031 (5.8) |
| F20.3^rd^.UC.Rz | Rep 1 | 23,025,180 | 97 | 19,866,720 (86.3) | 1,594,984 (6.9) | 1,563,476 (6.8) |
|  | Rep 2 | 23,318,471 | 95.9 | 20,053,190 (86) | 1,498,455 (6.4) | 1,766,826 (7.6) |
|  | Rep 3 | 25,045,450 | 96.5 | 21,439,740 (85.6) | 1,831,773 (7.3) | 1,773,937 (7.1) |

F20, fall 2020 harvest season; 1^st^, first harvest (22 September 2020); 2^nd^, second harvest (19 October 2020); 3^rd^, third harvest (05 November 2020); GM, cultivar ‘Guelph Millennium’; UC, cultivar ‘UC157’; Bu, dormant buds; Rz, rhizomes; Raw reads, total number of 100 bp paired-end raw reads obtained from sequencing on two lanes of Novaseq SP flowcell; Reads with Q score >30, Percentage of reads with phred quality score (Q score) greater than 30 (99.9% base call accuracy); Uniquely mapped reads, total number of reads mapping to single/unique loci; Multimapped reads, total number of reads mapping to multiple or too many loci; Unmapped reads, total number of unmapped reads including too many mismatches, too short, and others.

**Supplementary Table 4** Overview of sequencing and mapping of fall 2021 RNA-Seq samples in asparagus.

| **Sample** | **Biological replicate** | **Raw reads** | **Read percent with Q score > 30** | **Uniquely mapped reads (percent)** | **Multimapped reads (percent)** | **Unmapped reads (percent)** |
| --- | --- | --- | --- | --- | --- | --- |
| F21.1^st^.GM.Bu | Rep 1 | 25,111,638 | 97.1 | 21,820,082 (86.9) | 1,815,685 (7.2) | 1,475,871 (5.9) |
|  | Rep 2 | 23,435,623 | 97.8 | 20,557,298 (87.7) | 1,354,829 (5.8) | 1,523,496 (6.5) |
|  | Rep 3 | 30,043,932 | 97.2 | 26,272,330 (87.5) | 1,639,961 (5.4) | 2,131,641 (7.1) |
| F21.1^st^.GM.Rz | Rep 1 | 26,661,606 | 97.3 | 23,290,769 (87.4) | 1,388,270 (5.2) | 1,982,567 (7.4) |
|  | Rep 2 | 27,279,092 | 97.6 | 23,899,668 (87.6) | 1,341,896 (4.9) | 2,037,528 (7.5) |
|  | Rep 3 | 32,412,456 | 97.2 | 24,072,079 (74.3) | 6,120,464 (18.9) | 2,219,913 (6.8) |
| F21.2^nd^.GM.Bu | Rep 1 | 25,802,680 | 97.1 | 21,140,677 (81.9) | 3,050,482 (11.8) | 1,611,521 (6.3) |
|  | Rep 2 | 22,990,797 | 97.8 | 20,107,213 (87.5) | 1,430,009 (6.2) | 1,453,575 (6.3) |
|  | Rep 3 | 27,416,520 | 97.5 | 23,542,430 (85.9) | 2,295,318 (8.4) | 1,578,772 (5.7) |
| F21.2^nd^.GM.Rz | Rep 1 | 26,664,875 | 97.2 | 23,116,087 (86.7) | 1,530,957 (5.7) | 2,017,831 (7.6) |
|  | Rep 2 | 24,228,548 | 97.5 | 21,057,570 (86.9) | 1,243,198 (5.1) | 1,927,780 (8) |
|  | Rep 3 | 27,813,149 | 97.6 | 24,227,443 (87.1) | 1,486,379 (5.3) | 2,099,327 (7.6) |
| F21.3^rd^.GM.Bu | Rep 1 | 25,916,839 | 97.3 | 22,753,764 (87.8) | 1,514,271 (5.8) | 1,648,804 (6.4) |
|  | Rep 2 | 21,911,546 | 97.8 | 19,211,873 (87.7) | 1,200,370 (5.5) | 1,499,303 (6.8) |
|  | Rep 3 | 25,944,754 | 97.6 | 22,471,719 (86.6) | 1,666,274 (6.4) | 1,806,761 (7) |
| F21.3^rd^.GM.Rz | Rep 1 | 28,286,279 | 97.1 | 24,287,414 (85.9) | 1,780,911 (6.3) | 2,217,954 (7.8) |
|  | Rep 2 | 22,783,466 | 97.5 | 19,743,422 (86.7) | 1,268,315 (5.5) | 1,771,729 (7.8) |
|  | Rep 3 | 25,907,070 | 97.5 | 22,631,491 (87.4) | 1,325,267 (5.1) | 1,950,312 (7.5) |
| F21.1^st^.UC.Bu | Rep 1 | 24,897,612 | 97.3 | 21,722,222 (87.3) | 1,423,007 (5.7) | 1,752,383 (7) |
|  | Rep 2 | 25,588,828 | 97.1 | 22,401,253 (87.5) | 1,385,521 (5.4) | 1,802,054 (7.1) |
|  | Rep 3 | 30,394,138 | 97.1 | 26,498,545 (87.2) | 1,757,334 (5.8) | 2,138,259 (7) |
| F21.1^st^.UC.Rz | Rep 1 | 27,315,532 | 97.2 | 23,652,101 (86.6) | 1,288,598 (4.7) | 2,374,833 (8.7) |
|  | Rep 2 | 29,130,217 | 97.3 | 23,564,575 (80.9) | 3,331,757 (11.4) | 2,233,885 (7.7) |
|  | Rep 3 | 32,589,010 | 97.2 | 27,552,241 (84.5) | 2,591,370 (8) | 2,445,399 (7.5) |
| F21.2^nd^.UC.Bu | Rep 1 | 26,345,073 | 97 | 22,126,816 (84) | 2,440,080 (9.3) | 1,778,177 (6.7) |
|  | Rep 2 | 27,681,246 | 96.9 | 23,690,294 (85.6) | 2,264,726 (8.2) | 1,726,226 (6.2) |
|  | Rep 3 | 24,024,848 | 97.6 | 20,954,111 (87.2) | 1,577,142 (6.6) | 1,493,595 (6.2) |
| F21.2^nd^.UC.Rz | Rep 1 | 24,408,544 | 97.1 | 21,252,847 (87.1) | 1,234,330 (5) | 1,921,367 (7.9) |
|  | Rep 2 | 24,882,652 | 97.2 | 21,684,436 (87.1) | 1,259,098 (5.1) | 1,939,118 (7.8) |
|  | Rep 3 | 28,993,794 | 97.4 | 25,147,151 (86.7) | 1,716,415 (5.9) | 2,130,228 (7.4) |
| **Sample** | **Biological replicate** | **Raw reads** | **Read percent with Q score > 30** | **Uniquely mapped reads (percent)** | **Multimapped reads (percent)** | **Unmapped reads (percent)** |
| F21.3^rd^.UC.Bu | Rep 1 | 23,217,518 | 97.2 | 20,252,880 (87.2) | 1,338,646 (5.8) | 1,625,992 (7) |
|  | Rep 2 | 22,981,012 | 97.2 | 20,125,517 (87.6) | 1,248,471 (5.4) | 1,607,024 (7) |
|  | Rep 3 | 24,392,556 | 97.6 | 21,317,617 (87.4) | 1,398,136 (5.7) | 1,676,803 (6.9) |
| F21.3^rd^.UC.Rz | Rep 1 | 21,676,065 | 97.1 | 18,709,396 (86.3) | 1,089,442 (5) | 1,877,227 (8.7) |
|  | Rep 2 | 22,154,056 | 97.3 | 19,322,761 (87.2) | 1,126,575 (5.1) | 1,704,720 (7.7) |
|  | Rep 3 | 22,808,571 | 97.4 | 19,576,061 (85.8) | 1,230,341 (5.4) | 2,002,169 (8.8) |

F21, fall 2021 harvest season; 1^st^, first harvest (21 September 2021); 2^nd^, second harvest (13 October 2021); 3^rd^, third harvest (10 November 2021); GM, cultivar ‘Guelph Millennium’; UC, cultivar ‘UC157’; Bu, dormant buds; Rz, rhizomes; Raw reads, total number of 100 bp paired-end raw reads obtained from sequencing on two lanes of Novaseq SP flowcell; Reads with Q score >30, Percentage of reads with phred quality score (Q score) greater than 30 (99.9% base call accuracy); Uniquely mapped reads, total number of reads mapping to single/unique loci; Multimapped reads, total number of reads mapping to multiple or too many loci; Unmapped reads, total number of unmapped reads including too many mismatches, too short, and others.

Supplementary Table 5 Overview of sequencing and mapping of spring 2020 RNA-Seq samples in asparagus.

| **Sample** | **Biological replicate** | **Raw reads** | **Read percent with Q score > 30** | **Uniquely mapped reads (percent)** | **Multimapped reads (percent)** | **Unmapped reads (percent)** |
| --- | --- | --- | --- | --- | --- | --- |
| S20.1^st^.GM.Bu | Rep 1 | 23,064,370 | 98.6 | 20,138,365 (87.3) | 1,310,562 (5.7) | 1,615,443 (7) |
|  | Rep 2 | 21,416,055 | 98.8 | 18,291,150 (85.4) | 1,632,977 (7.6) | 1,491,928 (7) |
|  | Rep 3 | 23,192,314 | 98.6 | 19,718,967 (85) | 1,852,897 (8) | 1,620,450 (67) |
| S20.1^st^.GM.Rz | Rep 1 | 23,875,455 | 98.5 | 20,799,454 (87.1) | 1,236,145 (5.2) | 1,839,856 (7.7) |
|  | Rep 2 | 30,207,469 | 98.6 | 25,007,501 (82.8) | 3,019,077 (10) | 2,180,891 (7.2) |
|  | Rep 3 | 20,979,638 | 98.6 | 18,196,994 (86.7) | 1,136,518 (5.4) | 1,646,126 (7.8) |
| S20.2^nd^.GM.BL | Rep 1 | 19,619,148 | 98.6 | 15,198,673 (77.5) | 2,834,722 (14.5) | 1,585,753 (8.1) |
|  | Rep 2 | 36,088,624 | 98.2 | 31,083,756 (86.1) | 2,033,285 (5.6) | 2,971,583 (8.2) |
|  | Rep 3 | 25,218,364 | 98.3 | 20,896,277 (82.9) | 2,220,422 (8.8) | 2,101,665 (8.3) |
| S20.2^nd^.GM.Bu | Rep 1 | 21,820,628 | 98.4 | 19,054,599 (87.3) | 1,214,511 (5.6) | 1,551,518 (7.1) |
|  | Rep 2 | 17,795,806 | 98.6 | 15,237,074 (85.6) | 1,339,615 (7.5) | 1,219,117 (6.9) |
|  | Rep 3 | 24,075,801 | 98.7 | 20,673,692 (85.9) | 1,633,190 (6.8) | 1,768,919 (7.3) |
| S20.2^nd^.GM.Rz | Rep 1 | 20,922,568 | 98.5 | 18,231,760 (87.1) | 1,117,541 (5.3) | 1,573,267 (7.5) |
|  | Rep 2 | 20,118,055 | 98.3 | 17,387,455 (86.4) | 1,036,153 (5.2) | 1,694,447 (8.4) |
|  | Rep 3 | 23,921,947 | 98.6 | 20,031,681 (83.7) | 2,091,117 (8.7) | 1,799,149 (7.5) |
| S20.3^rd^.GM.BL | Rep 1 | 17,358,848 | 98.3 | 15,118,409 (87.1) | 853,073 (4.9) | 1,387,366 (7) |
|  | Rep 2 | 22,789,421 | 98.4 | 19,097,780 (83.8) | 2,116,170 (9.3) | 1,575,471 (6.9) |
|  | Rep 3 | 19,395,993 | 98.5 | 16,551,985 (85.3) | 1,428,718 (7.4) | 1,415,290 (7.3) |
| S20.3^rd^.GM.Rz | Rep 1 | 26,310,568 | 98.3 | 22,945,648 (87.2) | 1,471,515 (5.6) | 1,893,405 (7.2) |
|  | Rep 2 | 21,510,480 | 98.5 | 18,480,674 (85.9) | 1,359,675 (6.3) | 1,670,131 (7.8) |
|  | Rep 3 | 21,296,655 | 98.5 | 18,559,873 (87.2) | 1,275,409 (5.9) | 1,461,373 (6.9) |
| S20.1^st^.UC.BL | Rep 1 | 19,877,275 | 98.8 | 17,239,352 (86.7) | 1,313,001 (6.6) | 1,324,922 (6.7) |
|  | Rep 2 | 22,485,397 | 98.5 | 19,518,728 (86.8) | 1,414,403 (6.3) | 1,552,266 (6.9) |
|  | Rep 3 | 20,487,697 | 98.4 | 17,665,056 (86.2) | 1,170,066 (5.7) | 1,652,575 (8.1) |
| S20.1^st^.UC.Bu | Rep 1 | 23,627,403 | 98.7 | 19,569,849 (82.8) | 2,475,359 (10.5) | 1,582,195 (6.7) |
|  | Rep 2 | 18,666,541 | 98.8 | 12,716,451 (68.1) | 4,904,033 (26.3) | 1,046,057 (5.6) |
|  | Rep 3 | 21,971,290 | 98.5 | 19,044,317 (86.7) | 1,215,817 (5.5) | 1,711,156 (7.8) |
| S20.1^st^.UC.Rz | Rep 1 | 24,096,460 | 98.7 | 18,815,363 (78.1) | 3,610,945 (14) | 1,670,152 (6.9) |
|  | Rep 2 | 23,645,228 | 98.6 | 19,063,170 (80.6) | 2,547,003 (10.8) | 2,035,055 (8.6) |
|  | Rep 3 | 23,495,571 | 98.5 | 20,305,491 (86.4) | 1,222,921 (5.2) | 1,967,159 (8.4) |
| **Sample** | **Biological replicate** | **Raw reads** | **Read percent with Q score > 30** | **Uniquely mapped reads (percent)** | **Multimapped reads (percent)** | **Unmapped reads (percent)** |
| S20.2^nd^.UC.BL | Rep 1 | 22,636,316 | 98.5 | 19,294,538 (85.2) | 1,188,723 (5.3) | 2,153,055 (9.5) |
|  | Rep 2 | 28,177,698 | 98.4 | 24,211,241 (85.9) | 1,483,531 (5.3) | 2,482,926 (8.8) |
|  | Rep 3 | 26,235,486 | 98.3 | 22,163,059 (84.5) | 1,642,553 (6.3) | 2,429,874 (9.3) |
| S20.2^nd^.UC.Bu | Rep 1 | 22,469,281 | 98.5 | 18,611,070 (82.8) | 2,126,278 (9.5) | 1,731,933 (7.7) |
|  | Rep 2 | 22,056,363 | 98.6 | 19,082,799 (86.5) | 1,268,237 (5.8) | 1,705,327 (7.7) |
|  | Rep 3 | 24,959,000 | 98.4 | 20,672,385 (82.8) | 2,484,901 (9.0) | 1,801,714 (7.2) |
| S20.2^nd^.UC.Rz | Rep 1 | 24,825,104 | 98.3 | 21,029,892 (84.7) | 1,722,028 (6.9) | 2,073,184 (8.4) |
|  | Rep 2 | 25,109,943 | 98.5 | 21,654,388 (86.2) | 1,310,462 (5.2) | 2,145,093 (8.6) |
|  | Rep 3 | 24,416,563 | 98.3 | 21,076,408 (86.3) | 1,516,498 (6.2) | 1,823,657 (7.5) |
| S20.3^rd^.UC.BL | Rep 1 | 21,135,540 | 98.4 | 18,324,804 (86.7) | 1,085,794 (5.1) | 1,724,942 (8.2) |
|  | Rep 2 | 21,081,320 | 98.3 | 17,405,858 (82.6) | 2,193,467 (10.4) | 1,481,995 (7) |
|  | Rep 3 | 20,968,528 | 98.6 | 17,257,373 (82.3) | 2,290,185 (10.9) | 1,420,970 (6.8) |
| S20.3^rd^.UC.Rz | Rep 1 | 23,776,443 | 98.5 | 20,616,669 (86.7) | 1,505,039 (6.3) | 1,654,735 (7) |
|  | Rep 2 | 22,556,809 | 98.4 | 18,751,382 (83.1) | 2,177,282 (9.7) | 1,628,145 (7.2) |
|  | Rep 3 | 23,656,733 | 98.5 | 17,349,594 (73.3) | 4,863,549 (20.6) | 1,443,590 (6.1) |

S20, spring 2020 harvest season; 1^st^, first harvest (09 March 2020); 2^nd^, second harvest (25 March 2020); 3^rd^, third harvest (06 April 2020); GM, cultivar ‘Guelph Millennium’; UC, cultivar ‘UC157’; Bu, dormant buds; BL, growing buds; Rz, rhizomes; Raw reads, total number of 100 bp paired-end raw reads obtained from sequencing on two lanes of Novaseq SP flowcell; Reads with Q score >30, Percentage of reads with phred quality score (Q score) greater than 30 (99.9% base call accuracy); Uniquely mapped reads, total number of reads mapping to single/unique loci; Multimapped reads, total number of reads mapping to multiple or too many loci; Unmapped reads, total number of unmapped reads including too many mismatches, too short, and others.

**Supplementary Table 6** Overview of sequencing and mapping of spring 2021 RNA-Seq samples in asparagus.

| **Sample** | **Biological replicate** | **Raw reads** | **Read percent with Q score > 30** | **Uniquely mapped reads (percent)** | **Multimapped reads (percent)** | **Unmapped reads (percent)** |
| --- | --- | --- | --- | --- | --- | --- |
| S21.1^st^.GM.Bu | Rep 1 | 29,679,777 | 97.6 | 26,140,695 (88.1) | 1,707,178 (5.8) | 1,831,904 (6.2) |
|  | Rep 2 | 26,725,243 | 97.5 | 23,325,226 (87.3) | 1,589,321 (5.9) | 1,810,696 (6.8) |
|  | Rep 3 | 68,854,077 | 97.1 | 60,167,293 (87.4) | 3,625,614 (5.3) | 5,061,170 (7.3) |
| S21.1^st^.GM.Rz | Rep 1 | 28,378,892 | 97.5 | 24,530,693 (86.4) | 1,849,656 (6.5) | 1,998,543 (7.1) |
|  | Rep 2 | 30,882,454 | 97.6 | 27,147,837 (87.9) | 1,521,814 (4.9) | 2,212,803 (7.2) |
|  | Rep 3 | 25,820,106 | 97.4 | 22,215,956 (86.1) | 1,451,698 (5.6) | 2,152,452 (8.3) |
| S21.2^nd^.GM.BL | Rep 1 | 28,919,111 | 97.2 | 25,088,083 (86.8) | 1,382,881 (4.8) | 2,448,147 (8.5) |
|  | Rep 2 | 26,242,060 | 97.1 | 22,715,789 (86.6) | 1,354,362 (5.2) | 2,171,909 (8.3) |
|  | Rep 3 | 27,109,698 | 97.2 | 23,542,908 (86.8) | 1,342,637 (5) | 2,224,153 (8.2) |
| S21.2^nd^.GM.Bu | Rep 1 | 26,751,077 | 97.3 | 23,377,606 (87.4) | 1,552,146 (5.8) | 1,821,325 (6.8) |
|  | Rep 2 | 22,802,232 | 97.7 | 20,141,367 (88.3) | 1,230,992 (5.4) | 1,429,873 (6.3) |
|  | Rep 3 | 28,363,595 | 97.3 | 24,673,691 (87) | 1,726,148 (6.1) | 1,963,756 (6.9) |
| S21.2^nd^.GM.Rz | Rep 1 | 26,490,595 | 97.4 | 22,996,435 (86.8) | 1,446,467 (5.5) | 2,047,693 (7.7) |
|  | Rep 2 | 58,199,143 | 97 | 50,259,280 (86.4) | 3,886,338 (6.7) | 4,053,525 (6.9) |
|  | Rep 3 | 28,879,923 | 97.3 | 25,084,329 (86.9) | 1,468,657 (5) | 2,326,937 (8.1) |
| S21.3^rd^.GM.BL | Rep 1 | 23,550,015 | 97.6 | 20,568,312 (87.3) | 1,165,816 (5) | 1,815,887 (7.7) |
|  | Rep 2 | 30,069,377 | 97.4 | 26,097,952 (86.8) | 1,487,162 (4.9) | 2,484,263 (8.3) |
|  | Rep 3 | 31,116,419 | 97.4 | 27,183,875 (87.4) | 1439,367 (4.6) | 2,493,177 (8.) |
| S21.3^rd^.GM.Rz | Rep 1 | 60,971,673 | 97.1 | 52,673,904 (86.4) | 3,338,995 (5.5) | 4,958,774 (8.1) |
|  | Rep 2 | 28,539,501 | 97.6 | 24,499,604 (85.8) | 1,408,385 (4.9) | 2,631,512 (9.2) |
|  | Rep 3 | 36,208,271 | 97.6 | 31,269,413 (86.4) | 1,944,115 (5.4) | 2,994,743 (8.2) |
| S21.1^st^.UC.BL | Rep 1 | 28,744,179 | 97.4 | 24,761,432 (86.1) | 1,581,797 (5.5) | 2,400,950 (8.4) |
|  | Rep 2 | 19,834,872 | 97.7 | 17,363,188 (87.5) | 996,667 (5) | 1,475,017 (7.4) |
|  | Rep 3 | 30,194,488 | 97.3 | 26,332,538 (87.2) | 1,446,892 (4.8) | 2,415,058 (8) |
| S21.1^st^.UC.Bu | Rep 1 | 28,128,225 | 97.5 | 24,574,255 (87.4) | 1,367,525 (4.8) | 2,186,445 (7.8) |
|  | Rep 2 | 26,074,670 | 97.7 | 22,808,853 (87.5) | 1,335,670 (5.1) | 1,930,147 (7.4) |
|  | Rep 3 | 31,595,660 | 97.5 | 27,340,522 (86.5) | 1,586,015 (5) | 2,669,123 (8.44) |
| S21.1^st^.UC.Rz | Rep 1 | 25,999,269 | 97.4 | 22,091,753 (85) | 1,405,046 (5.4) | 2,502,470 (9.6) |
|  | Rep 2 | 29,595,627 | 97.4 | 25,235,282 (85.3) | 1,482,727 (5) | 2,877,618 (9.7) |
|  | Rep 3 | 30,394,862 | 97.3 | 26,097,618 (85.9) | 1,469,760 (4.8) | 2,827,484 (9.3) |
| **Sample** | **Biological replicate** | **Raw reads** | **Read percent with Q score > 30** | **Uniquely mapped reads (percent)** | **Multimapped reads (percent)** | **Unmapped reads (percent)** |
| S21.2^nd^.UC.BL | Rep 1 | 30,300,515 | 97.4 | 26,289,811 (86.8) | 1,497,956 (4.9) | 2,512,748 (8.3) |
|  | Rep 2 | 29,341,342 | 97.3 | 25,484,797 (86.9) | 1,485,530 (5.1) | 2,371,015 (8.1) |
|  | Rep 3 | 32,574,381 | 97.2 | 27,187,018 (83.5) | 2,827,404 (8.7) | 2,559,959 (7.8) |
| S21.2^nd^.UC.Bu | Rep 1 | 30,334,346 | 97.4 | 26,403,753 (87) | 1,591,017 (5.3) | 2,339,576 (7.7) |
|  | Rep 2 | 31,491,187 | 97.6 | 27,607,127 (87.7) | 1,615,342 (5.1) | 2,268,718 (7.2) |
|  | Rep 3 | 31,319,948 | 97.4 | 26,979,394 (86.1) | 2,028,896 (6.5) | 2,311,658 (7.4) |
| S21.2^nd^.UC.Rz | Rep 1 | 30,666,677 | 97.6 | 26,405,845 (86.1) | 1,580,356 (5.2) | 2,680,476 (8.7) |
|  | Rep 2 | 30,030,649 | 97.3 | 25,514,021 (85) | 1,508,857 (5) | 3,007,771 (10) |
|  | Rep 3 | 30,716,273 | 97.6 | 26,645,942 (86.8) | 1,641,162 (5.3) | 2,429,169 (7.9) |
| S21.3^rd^.UC.BL | Rep 1 | 26,222,484 | 97.2 | 22,735,420 (86.7) | 1,297,560 (4.9) | 2,189,504 (8.3) |
|  | Rep 2 | 26,944,876 | 97.4 | 23,517,084 (87.3) | 1,235,113 (4.6) | 2,192,679 (8.1) |
|  | Rep 3 | 31,586,204 | 97.7 | 27,345,545 (86.6) | 1,591,409 (5) | 2,649,250 (8.4) |
| S21.3^rd^.UC.Rz | Rep 1 | 38,814,899 | 97.8 | 32,967,129 (84.9) | 2,309,204 (6) | 3,538,566 (9.1) |
|  | Rep 2 | 32,751,763 | 97.6 | 28,648,138 (87.5) | 1,694,980 (5.2) | 2,408,645 (7.3) |
|  | Rep 3 | 35,783,061 | 97.5 | 30,840,368 (86.2) | 1,817,592 (5.1) | 3,125,101 (8.7) |

S21, spring 2021 harvest season; 1^st^, first harvest (22 March 2021); 2^nd^, second harvest (29 March 2021); 3^rd^, third harvest (14 April 2021); GM, cultivar ‘Guelph Millennium’; UC, cultivar ‘UC157’; Bu, dormant buds; BL, growing buds; Rz, rhizomes; Raw reads, total number of 100 bp paired-end raw reads obtained from sequencing on two lanes of Novaseq SP flowcell; Reads with Q score >30, Percentage of reads with phred quality score (Q score) greater than 30 (99.9% base call accuracy); Uniquely mapped reads, total number of reads mapping to single/unique loci; Multimapped reads, total number of reads mapping to multiple or too many loci; Unmapped reads, total number of unmapped reads including too many mismatches, too short, and others.

Supplementary Table 7 Number of differentially expressed (DE) genes at the second and third harvests relative to first harvest within dormant buds (Bu) and rhizomes (Rz) of asparagus cultivars ‘Guelph Millennium’ (GM) and ‘UC157’ (UC) during the fall 2020 season.

| **Samples compared** | **DE genes** | **Downregulated genes (percent)** | **Upregulated genes (percent)** |
| --- | --- | --- | --- |
| **Dormant buds** | | | |
| 2^nd^.GM.Bu vs 1^st^.GM.Bu | 1,037 | 674 (65) | 363 (35) |
| 3^rd^.GM.Bu vs 1^st^.GM.Bu | 1,265 | 1,130 (89.3) | 135 (10.7) |
| 2^nd^.UC.Bu vs 1^st^.UC.Bu | 237 | 164 (69.2) | 73 (30.8) |
| 3^rd^.UC.Bu vs 1^st^.UC.Bu | 648 | 549 (84.7 | 99 (15.3) |
| **Rhizomes** | | | |
| 2^nd^.GM.Rz vs 1^st^.GM.Rz | 971 | 628 (64.7) | 343 (35.3) |
| 3^rd^.GM.Rz vs 1^st^.GM.Rz | 1,186 | 891 (75.1) | 295 (24.9) |
| 2^nd^.UC.Rz vs 1^st^.UC.Rz | 861 | 537 (62.4) | 324 (37.6) |
| 3^rd^.UC.Rz vs 1^st^.UC.Rz | 1,191 | 870 (73.1) | 321 (27) |

DE genes, total number of differentially expressed genes based on the adjusted *p*-value i.e., false discovery rate of less than 0.05 and log_2_fold change difference of greater than 2; 1^st^, first harvest (22 September 2020); 2^nd^, second harvest (19 October 2020); 3^rd^, third harvest (05 November 2020).

Supplementary Table 8 Number of differentially expressed (DE) genes at the second and third harvests relative to first harvest within dormant buds (Bu) and rhizomes (Rz) of asparagus cultivars ‘Guelph Millennium’ (GM) and ‘UC157’ (UC) during the fall 2021 season.

| **Samples compared** | **DE genes** | **Downregulated genes (percent)** | **Upregulated genes (percent)** |
| --- | --- | --- | --- |
| **Dormant buds** | | | |
| 2^nd^.GM.Bu vs 1^st^.GM.Bu | 102 | 84 (82.4) | 18 (17.6) |
| 3^rd^.GM.Bu vs 1^st^.GM.Bu | 1,354 | 1,174 (86.7) | 180 (13.3) |
| 2^nd^.UC.Bu vs 1^st^.UC.Bu | 24 | 19 (79.2) | 5 (20.8) |
| 3^rd^.UC.Bu vs 1^st^.UC.Bu | 1,287 | 1,103 (85.7) | 184 (14.3) |
| **Rhizomes** | | | |
| 2^nd^.GM.Rz vs 1^st^.GM.Rz | 190 | 113 (59.5) | 77 (40.5) |
| 3^rd^.GM.Rz vs 1^st^.GM.Rz | 2,036 | 1,365 (67) | 671 (33) |
| 2^nd^.UC.Rz vs 1^st^.UC.Rz | 48 | 22 (45.8) | 26 (54.2) |
| 3^rd^.UC.Rz vs 1^st^.UC.Rz | 2,004 | 1,478 (73.8) | 526 (26.2) |

DE genes, total number of differentially expressed (DE) genes based on the adjusted *p*-value i.e., false discovery rate of less than 0.05 and log_2_fold change difference of greater than 2; 1^st^, first harvest (21 September 2021); 2^nd^, second harvest (13 October 2021); 3^rd^, third harvest (10 November 2021).

Supplementary Table 9 Number of differentially expressed (DE) genes at the second and third harvests relative to first harvest within dormant buds (Bu), growing buds (BL), and rhizomes (Rz) of asparagus cultivars ‘Guelph Millennium’ (GM) and ‘UC157’ (UC) during the spring 2020 season.

| **Samples compared** | **DE genes** | **Downregulated genes (percent)** | **Upregulated genes (percent)** |
| --- | --- | --- | --- |
| **Dormant and growing buds** | | | |
| 2^nd^.GM.Bu vs 1^st^.GM.Bu | 656 | 153 (23.3) | 503 (76.7) |
| 2^nd^.GM.BL vs 1^st^.GM.Bu | 1,642 | 320 (19.5) | 1,322 (80.5) |
| 3^rd^.GM.BL vs 1^st^.GM.Bu | 1,964 | 472 (24) | 1,492 (76) |
| 1^st^.UC.BL vs 1^st^.UC.Bu | 56 | 28 (50) | 28 (50) |
| 2^nd^.UC.Bu vs 1^st^.UC.Bu | 704 | 165 (23.4) | 539 (76.6) |
| 2^nd^.UC.BL vs 1^st^.UC.Bu | 1,534 | 399 (26) | 1,135 (74) |
| 3^rd^.UC.BL vs 1^st^.UC.Bu | 1,945 | 537 (27.6) | 1,408 (72.4) |
| **Rhizomes** | | | |
| 2^nd^.GM.Rz vs 1^st^.GM.Rz | 335 | 65 (19.4) | 270 (80.6) |
| 3^rd^.GM.Rz vs 1^st^.GM.Rz | 967 | 375 (38.8) | 592 (61.2) |
| 2^nd^.UC.Rz vs 1^st^.UC.Rz | 259 | 76 (29.3) | 183 (70.7) |
| 3^rd^.UC.Rz vs 1^st^.UC.Rz | 1,277 | 442 (34.6) | 835 (65.4) |

DE genes, total number of differentially expressed genes based on the adjusted *p*-value i.e., false discovery rate of less than 0.05 and log_2_fold change difference of greater than 2; 1^st^, first harvest (09 March 2020); 2^nd^, second harvest (25 March 2020); 3^rd^, third harvest (06 April 2020).

Supplementary Table 10 Number of differentially expressed (DE) genes at the second and third harvests relative to first harvest within dormant buds (Bu), growing buds (BL), and rhizomes (Rz) of asparagus cultivars ‘Guelph Millennium’ (GM) and ‘UC157’ (UC) during the spring 2021 season.

| **Samples compared** | **DE genes** | **Downregulated genes (percent)** | **Upregulated genes (percent)** |
| --- | --- | --- | --- |
| **Dormant and growing buds** | | | |
| 2^nd^.GM.Bu vs 1^st^.GM.Bu | 29 | 8 (27.6) | 21 (72.4) |
| 2^nd^.GM.BL vs 1^st^.GM.Bu | 1,071 | 128 (12) | 943 (88) |
| 3^rd^.GM.BL vs 1^st^.GM.Bu | 1,563 | 190 (12.2) | 1,373 (87.8) |
| 1^st^.UC.BL vs 1^st^.UC.Bu | 131 | 30 (22.9) | 101 (77.1) |
| 2^nd^.UC.Bu vs 1^st^.UC.Bu | 67 | 23 (34.3) | 44 (65.7) |
| 2^nd^.UC.BL vs 1^st^.UC.Bu | 755 | 100 (13.2) | 655 (86.8) |
| 3^rd^.UC.BL vs 1^st^.UC.Bu | 1,442 | 145 (10.1) | 1,297 (89.9) |
| **Rhizomes** | | | |
| 2^nd^.GM.Rz vs 1^st^.GM.Rz | 381 | 53 (13.9) | 328 (86.1) |
| 3^rd^.GM.Rz vs 1^st^.GM.Rz | 1,439 | 243 (16.9) | 1,196 (83.1) |
| 2^nd^.UC.Rz vs 1^st^.UC.Rz | 170 | 52 (30.6) | 118 (69.4) |
| 3^rd^.UC.Rz vs 1^st^.UC.Rz | 1,161 | 319 (27.5) | 842 (72.5) |

DE genes, total number of differentially expressed (DE) genes based on the adjusted *p*-value i.e., false discovery rate of less than 0.05 and log_2_fold change difference of greater than 2; 1^st^, first harvest (22 March 2021); 2^nd^, second harvest (29 March 2021); 3^rd^, third harvest (14 April 2021).

Supplementary Table 11 Summary of GO terms and metabolic pathways assigned to genes differentially expressed in the dormant buds (Bu), growing buds (BL), and rhizomes (Rz) of asparagus cultivars ‘Guelph Millennium’ (GM) and ‘UC157’ (UC) during fall acclimation and spring deacclimation.

| **GO terms/metabolic pathways** | **Consistent with LT_50_** | | | | | | **Inconsistent with LT_50_** | | | | | |
| --- | --- | --- | --- | --- | --- | --- | --- | --- | --- | --- | --- | --- |
|  | **Down in fall** | | **Up in spring** | **Up in fall** | | **Down in spring** | **Down in fall** | | **Up in spring** | **Up in fall** | | **Down in spring** |
|  | **Bu** | **Rz** | **BL and Rz** | **Bu** | **Rz** | **BL and Rz** | **Bu** | **Rz** | **BL and Rz** | **Bu** | **Rz** | **BL and Rz** |
| GO:0005975~carbohydrate metabolic process | + | + | + | + |  |  |  |  |  |  | + | + |
| GO:0006979~response to oxidative stress | + | + | + |  |  |  |  |  |  |  |  |  |
| GO:0042744~hydrogen peroxide catabolic process | + | + | + |  |  |  |  |  |  |  |  |  |
| GO:0042545/0071555~cell wall modification/organization | + | + | + |  |  |  |  |  |  |  |  |  |
| GO:0006629~lipid metabolic process | + | + | + |  |  |  |  |  |  |  |  |  |
| GO:0046274~lignin catabolic process | + | + | + |  |  |  |  |  |  |  |  |  |
| GO:0006865~amino acid transport | + | + | + |  |  |  |  |  |  |  |  |  |
| GO:0007018~microtubule-based movement | + | + |  |  |  |  |  |  | + |  |  |  |
| GO:0045490~pectin catabolic process | + | + | + |  |  |  |  |  |  |  |  |  |
| GO:0015979~photosynthesis | + |  | + |  |  |  |  |  |  |  |  |  |
| GO:0018298~protein-chromophore linkage | + |  | + |  |  |  |  |  |  |  |  |  |
| GO:0009765~photosynthesis, light harvesting | + |  | + |  |  |  |  |  |  |  |  |  |
| GO:0009409~response to cold | + |  | + |  |  |  |  |  |  |  |  |  |
| GO:0006749~glutathione metabolic process |  | + | + |  |  |  |  |  |  |  |  |  |
| GO:0016021~integral component of membrane | + | + | + | + | + | + |  |  |  |  |  |  |
| GO:0005576~extracellular region | + | + | + |  |  |  |  |  |  |  |  |  |
| GO:0005618~cell wall | + | + | + |  |  |  |  |  |  |  |  |  |
| GO:0048046~apoplast | + | + | + |  |  |  |  |  |  |  |  |  |
| GO:0005874~microtubule | + | + |  |  |  |  |  |  | + |  |  |  |
| GO:0005886~plasma membrane | + | + | + |  |  |  |  |  |  | + | + |  |
| GO:0005794~golgi apparatus | + | + | + |  |  |  |  |  |  |  |  |  |
| GO:0009523~photosystem II | + |  | + |  |  |  |  |  |  |  |  |  |
| GO:0009654~photosystem II oxygen evolving complex | + |  | + |  |  |  |  |  |  |  |  |  |
| GO:0009522~photosystem I | + |  | + |  |  |  |  |  |  |  |  |  |
| GO:0009538~photosystem I reaction center | + |  | + |  |  |  |  |  |  |  |  |  |
| GO:0009535~chloroplast thylakoid membrane | + |  | + |  |  |  |  |  |  |  |  |  |
| GO:0009507~chloroplast | + |  | + (BL) |  |  |  |  |  |  |  |  |  |
| **GO terms/metabolic pathways** | **Consistent with LT_50_** | | | | | | **Inconsistent with LT_50_** | | | | | |
|  | **Down in fall** | | **Up in spring** | **Up in fall** | | **Down in spring** | **Down in fall** | | **Up in spring** | **Up in fall** | | **Down in spring** |
|  | **Bu** | **Rz** | **BL and Rz** | **Bu** | **Rz** | **BL and Rz** | **Bu** | **Rz** | **BL and Rz** | **Bu** | **Rz** | **BL and Rz** |
| GO:0045492~xylan biosynthetic process |  |  | + |  |  |  | + | + |  |  |  |  |
| GO:0006869~lipid transport |  |  | + |  |  |  | + | + |  |  |  |  |
| GO:0007049/0051301~cell cycle/division |  |  |  |  |  |  | + | + | + |  |  |  |
| GO:0009873~ethylene-activated signalling pathway |  |  | + |  |  |  | + | + |  |  |  |  |
| GO:0009773~photosynthetic electron transport in photosystem I |  |  | + (BL) |  |  |  |  |  |  |  |  |  |
| GO:0009644~response to high light intensity |  |  | + (BL) |  |  |  |  |  |  |  |  |  |
| GO:0010207~photosystem II assembly |  |  | + (BL) |  |  |  |  |  |  |  |  |  |
| GO:0009800~cinnamic acid biosynthetic process |  |  | + |  |  |  |  |  |  |  |  |  |
| GO:0005992~trehalose biosynthetic process |  |  | + |  |  |  |  | + |  |  |  |  |
| GO:0071704~organic substance metabolic process |  |  |  |  |  |  |  | + |  |  |  |  |
| GO:0019898~extrinsic component of membrane |  |  | + |  |  |  | + |  |  |  |  |  |
| GO:0009570~chloroplast stroma |  |  | + (BL) |  |  |  |  |  |  |  |  |  |
| GO:0009941~chloroplast envelope |  |  | + (BL) |  |  |  |  |  |  |  |  |  |
| GO:0009579~thylakoid |  |  | + |  |  |  |  |  |  |  |  |  |
| GO:0031977~thylakoid lumen |  |  | + |  |  |  |  |  |  |  |  |  |
| GO:0000786~nucleosome |  |  |  |  |  |  |  |  | + |  |  |  |
| GO:0007623~circadian rhythm |  |  |  |  |  |  |  |  |  | + | + | + |
| GO:0009408~response to heat |  |  |  |  |  |  |  |  |  | + | + | + |
| GO:0048544~recognition of pollen |  |  |  |  |  |  |  |  |  | + | + |  |
| GO:0009415~response to water |  |  |  | + |  |  |  |  |  |  | + | + |
| GO:0005634~nucleus |  |  |  |  | + |  |  |  |  |  |  | + |
| aof01100~metabolic pathways | + | + | + |  |  |  |  |  |  |  |  |  |
| aof01110~biosynthesis of secondary metabolites | + | + | + |  |  |  |  |  |  |  |  |  |
| aof04075~plant hormone signal transduction | + | + | + |  |  |  |  |  |  |  |  | + |
| aof00940~phenylpropanoid biosynthesis | + | + | + |  |  |  |  |  |  | + |  | + |
| aof00500~starch and sucrose metabolism | + | + | + |  |  |  |  |  |  |  |  |  |
| aof00100~steroid biosynthesis | + | + | + |  |  |  |  |  |  |  |  |  |
| aof00941~flavonoid biosynthesis | + | + | + |  |  |  |  |  |  |  |  |  |
| aof00195~photosynthesis | + |  | + |  |  |  |  |  |  |  |  |  |
| aof00196~photosynthesis - antenna proteins | + |  | + |  |  |  |  |  |  |  |  |  |
| **GO terms/metabolic pathways** | **Consistent with LT_50_** | | | | | | **Inconsistent with LT_50_** | | | | | |
|  | **Down in fall** | | **Up in spring** | **Up in fall** | | **Down in spring** | **Down in fall** | | **Up in spring** | **Up in fall** | | **Down in spring** |
|  | **Bu** | **Rz** | **BL and Rz** | **Bu** | **Rz** | **BL and Rz** | **Bu** | **Rz** | **BL and Rz** | **Bu** | **Rz** | **BL and Rz** |
| aof00480~glutathione metabolism |  | + | + |  |  |  |  |  |  |  |  |  |
| aof00999~biosynthesis of various plant secondary metabolites |  |  | + |  |  |  | + | + |  |  |  |  |
| aof01200~carbon metabolism |  |  | + |  |  |  | + | + |  |  |  |  |
| aof00710~carbon fixation in photosynthetic organisms |  |  | + (BL) |  |  |  | + |  |  |  |  |  |
| aof01230~biosynthesis of amino acids |  |  |  |  |  |  |  | + |  |  |  |  |
| aof00400~phenylalanine, tyrosine and tryptophan biosynthesis |  |  |  |  |  |  |  | + |  |  |  |  |
| aof00330~arginine and proline metabolism |  |  | + |  |  |  | + |  |  |  |  |  |
| aof04141~protein processing in endoplasmic reticulum |  |  |  |  |  |  |  |  |  | + | + | + |
| aof00053~ascorbate and aldarate metabolism |  |  |  |  |  |  |  |  |  |  | + |  |
| aof02010~ABC transporters |  |  |  |  |  |  |  |  |  | + |  | + |
| aof00600~sphinigolipid metabolism |  |  |  |  |  |  |  |  |  |  |  | + |

Gene expression generalized over the second and third harvests of fall 2020, 2021 and spring 2020, 2021. ‘Consistent with LT_50_’ in fall indicates the number of genes assigned to each term/pathway were consistent either with greater LT_50_ values of GM than UC in fall 2020 (GM > UC) or equal LT_50_ values of both cultivars in fall 2021 (GM = UC). ‘Consistent with LT_50_’ in spring indicates the number of genes assigned to each term/pathway were consistent with equal LT_50_ values at third harvests either in dormant buds or rhizomes during 2020 or 2021. Down, downregulated GO terms or pathways. Up, upregulated GO terms or pathways. Bu, dormant buds; BL, growing buds; Rz, rhizomes. + (BL), upregulated only in the growing buds.

# Supplementary Figures


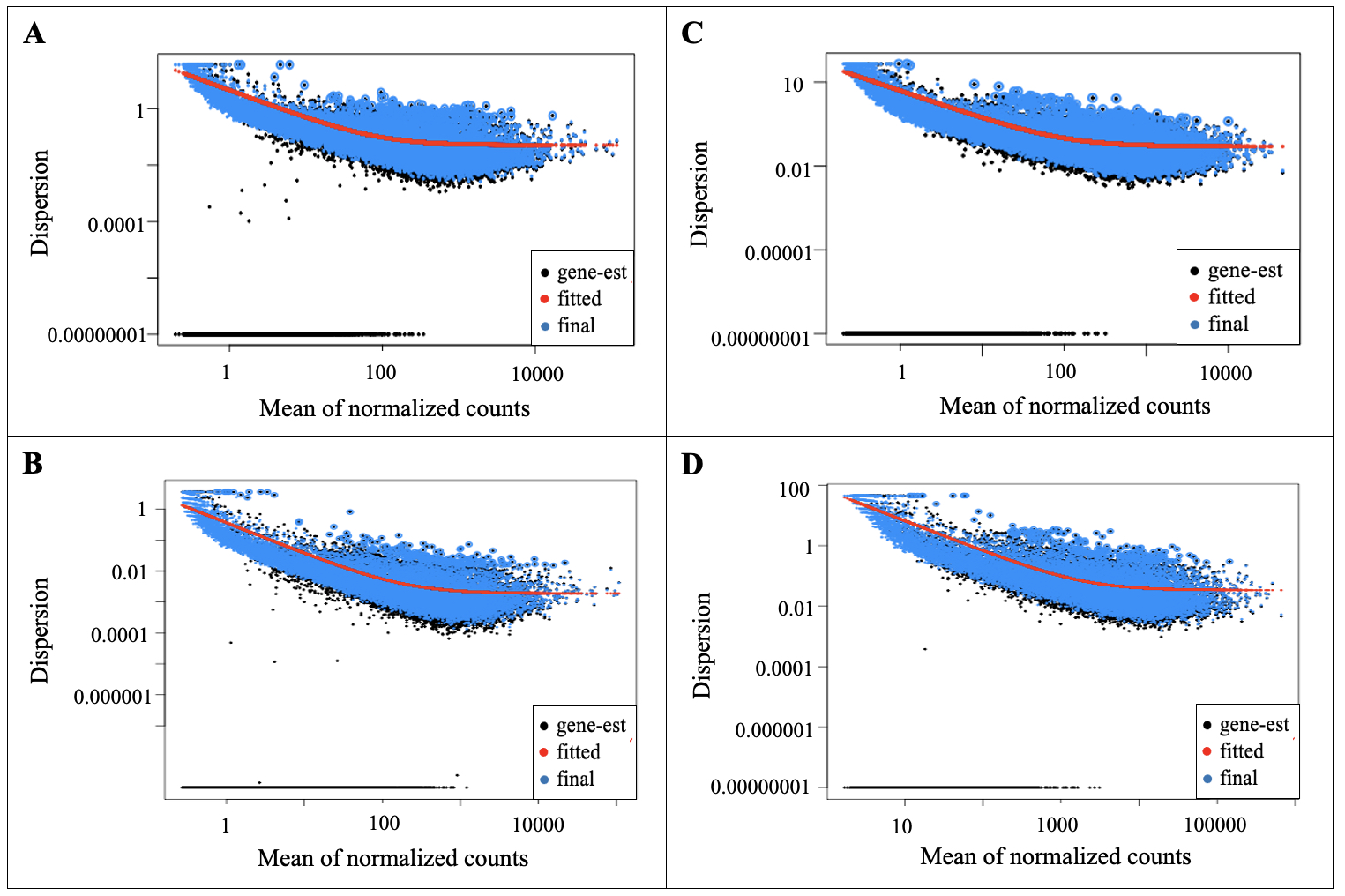


Supplementary Figure 1 Dispersion plots from modeling of normalized counts for differential gene expression analysis for (A) fall 2020, (B) fall 2021, (C) spring 2020, and (D) spring 2021 RNA-Seq data in asparagus. Mean of normalized counts (X-axis) were plotted against the dispersion estimates (Y-axis). Black dots represent the dispersion for each gene. Red line is the curve fitted to show the expected dispersion value for genes, and blue dots represent the shrunken gene-wise dispersion estimates toward the expected dispersion values.


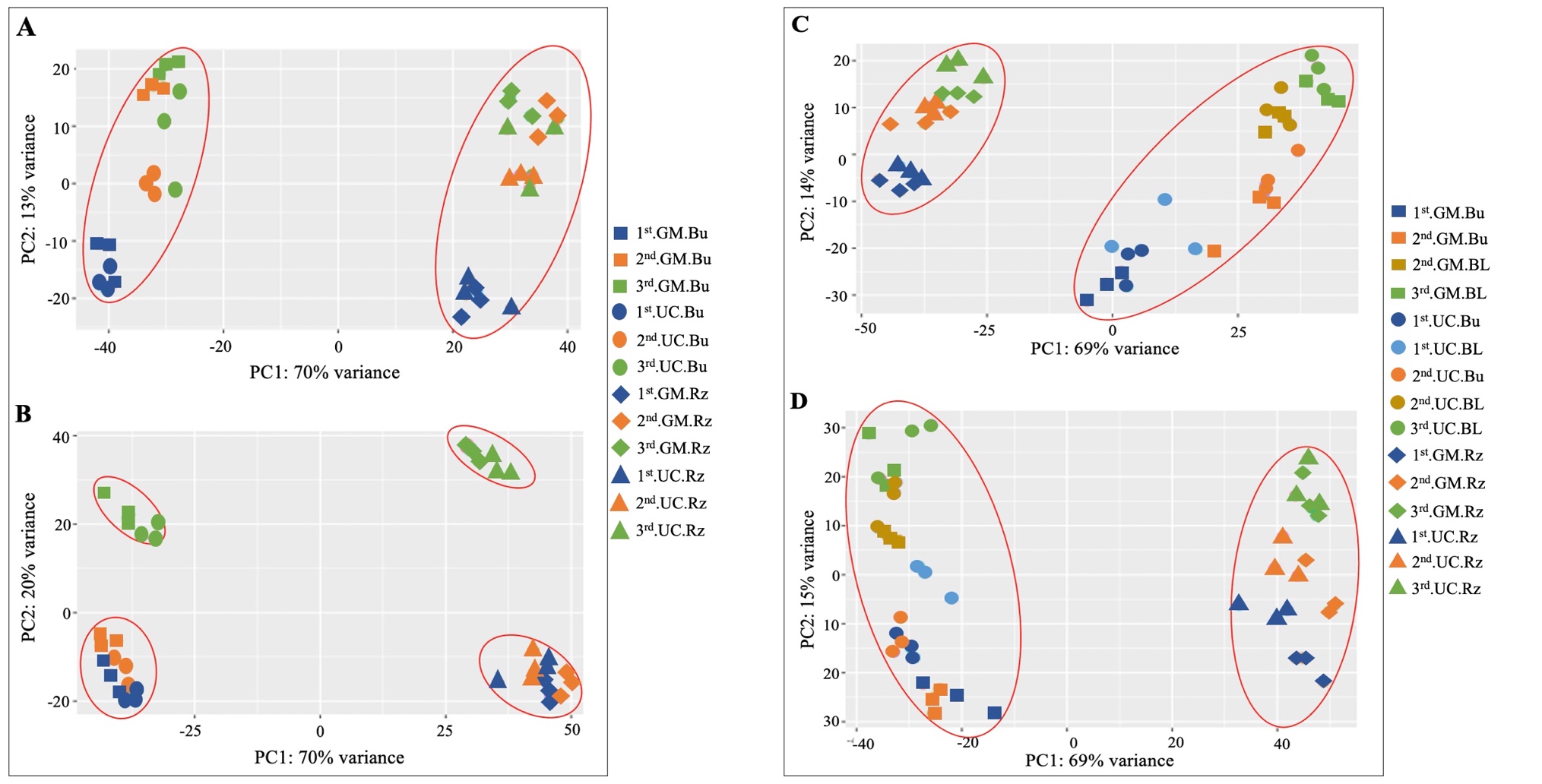


Supplementary Figure 2 Principal component analysis (PCA) plots of RNA-Seq data from dormant buds (Bu) and rhizomes (Rz) in (A) fall 2020 and (B) fall 2021 and from dormant buds, growing buds (BL), and rhizomes in (C) spring 2020 and (D) spring 2021 in asparagus cultivars ‘Guelph Millennium’ (GM) and ‘UC157’ (UC). Three dots of the same color represent the three biological replicates of each harvest date. 1^st^, first harvest; 2^nd^, second harvest; 3^rd^, third harvest.

**
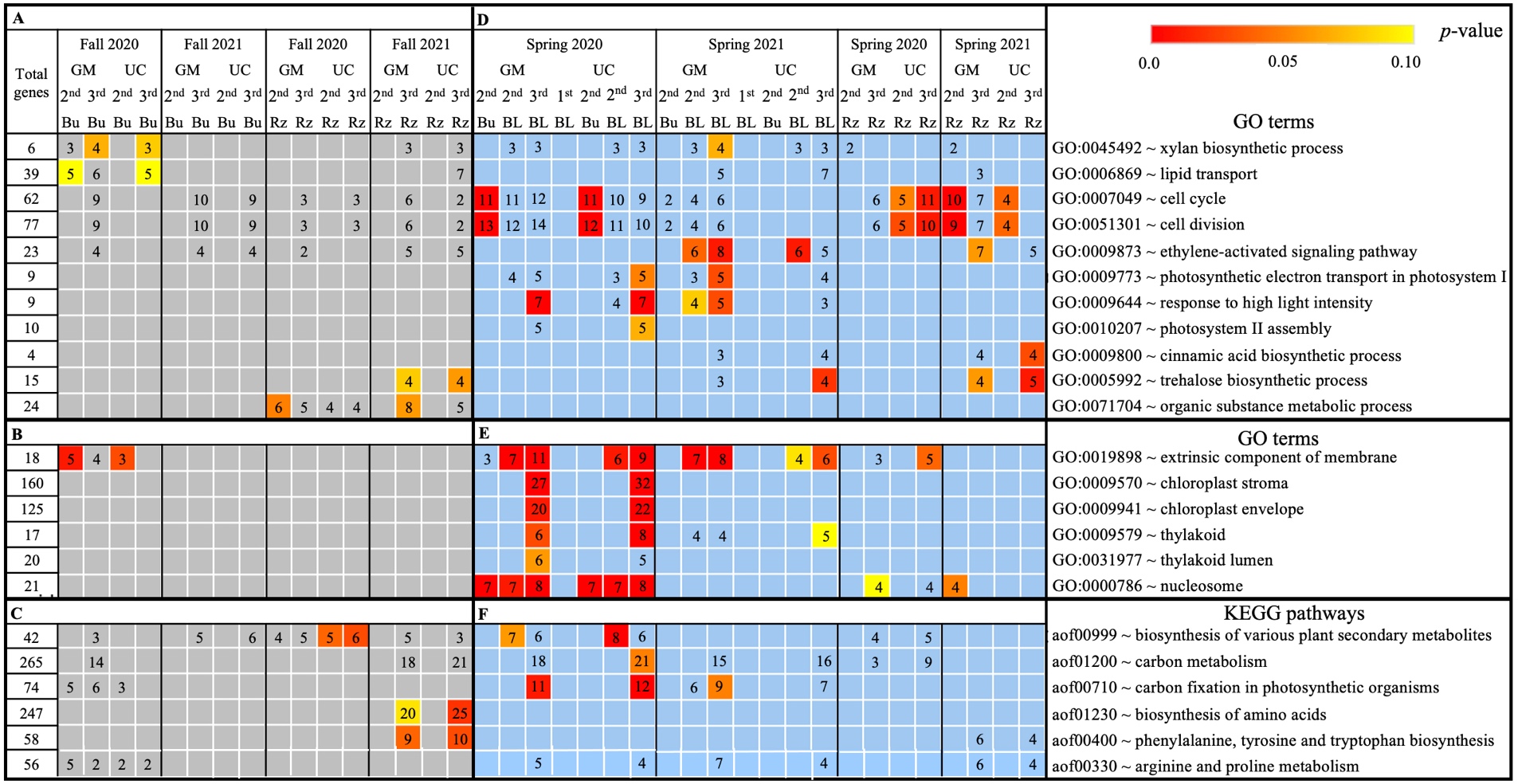
**

**Supplementary Figure 3** Gene ontology (GO) terms and metabolic pathways from KEGG database (inconsistent with LT_50_ patterns) assigned to genes differentially expressed (DE) relative to the first harvest for asparagus cultivars ‘Guelph Millennium’ (GM) and ‘UC157’ (UC). **(A)** Biological process GO terms, **(B)** Cellular component GO terms, and **(C)** KEGG pathways downregulated in dormant buds (Bu) and rhizomes (Rz) during the fall (2020 and 2021); **(D)** Biological process GO terms, **(E)** Cellular component GO terms, and **(F)** KEGG pathways upregulated in dormant buds, growing buds (BL), and rhizomes during the spring (2020 and 2021). Number of genes assigned to each GO term are presented in the cells; blank boxes equal zero. Within upregulated (blue area) and downregulated (grey area) genes, significantly enriched terms are color-coded based on an adjusted *p*-value i.e., false discovery rate of less than 0.1 (red most significant). 1^st^, first harvest; 2^nd^, second harvest; 3^rd^, third harvest.


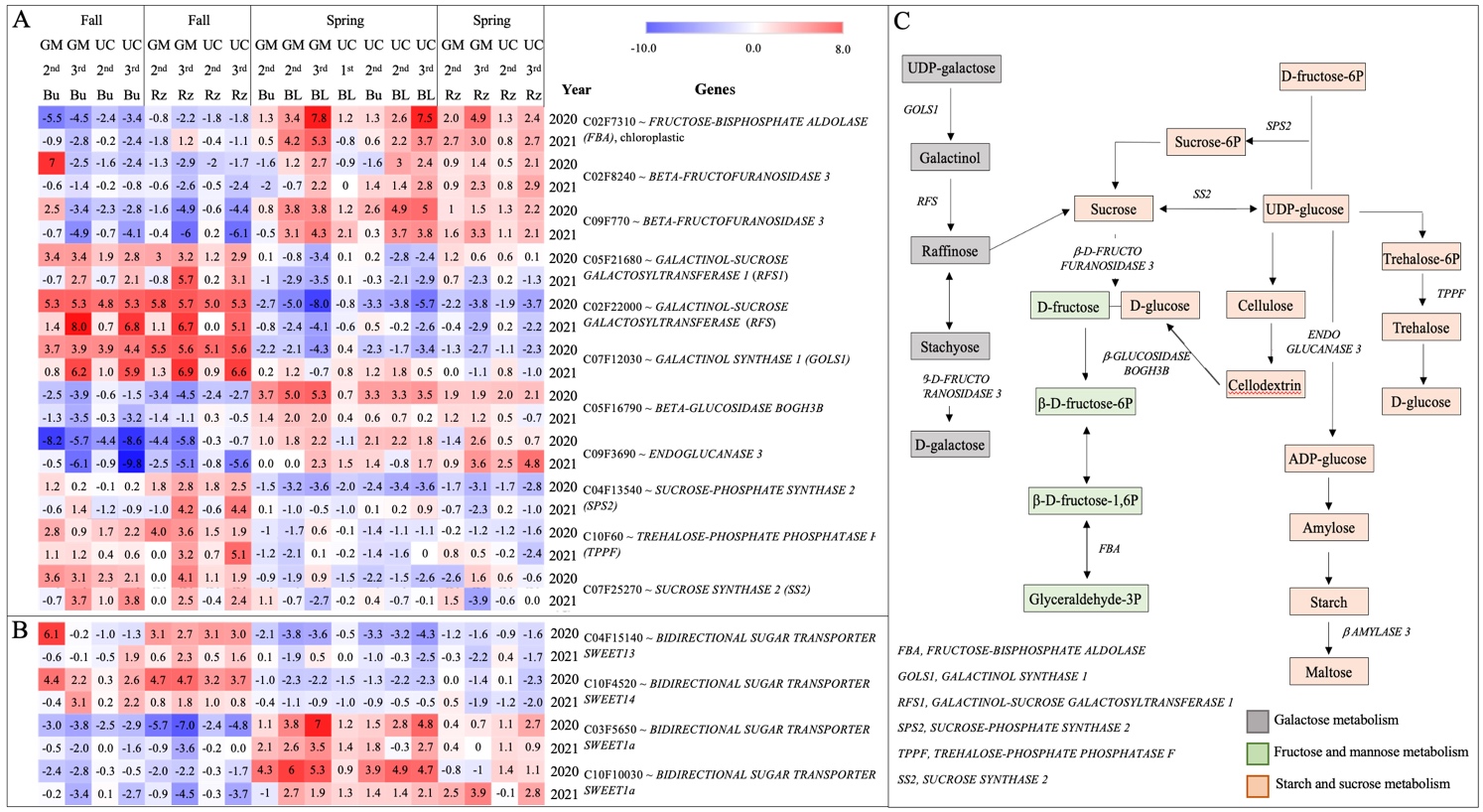


**Supplementary Figure 4** Expression patterns (log_2_fold differences) of differentially expressed genes involved in **(A)** fructose and mannose, galactose, and starch and sucrose metabolism and **(B)** sugar transport that showed expression patterns consistent with LT_50_ differences or similarities within dormant buds (Bu), growing buds (BL), and rhizomes (Rz) of asparagus cultivars ‘Guelph Millennium’ (GM) and ‘UC157’ (UC) during fall acclimation and spring deacclimation. **(C)** Fructose and mannose, galactose, and starch and sucrose metabolic pathways in asparagus to which differentially expressed genes were mapped. Pathways were modified from KEGG database (<http://www.kegg.jp>). Log_2_fold differences relative to dormant buds and rhizomes of first harvest are presented in the cells. Upregulated genes are color-coded red (log_2_fold difference > 0) and downregulated genes are color-coded blue (log_2_fold difference < 0). 1^st^, first harvest; 2^nd^, second harvest; 3^rd^, third harvest.


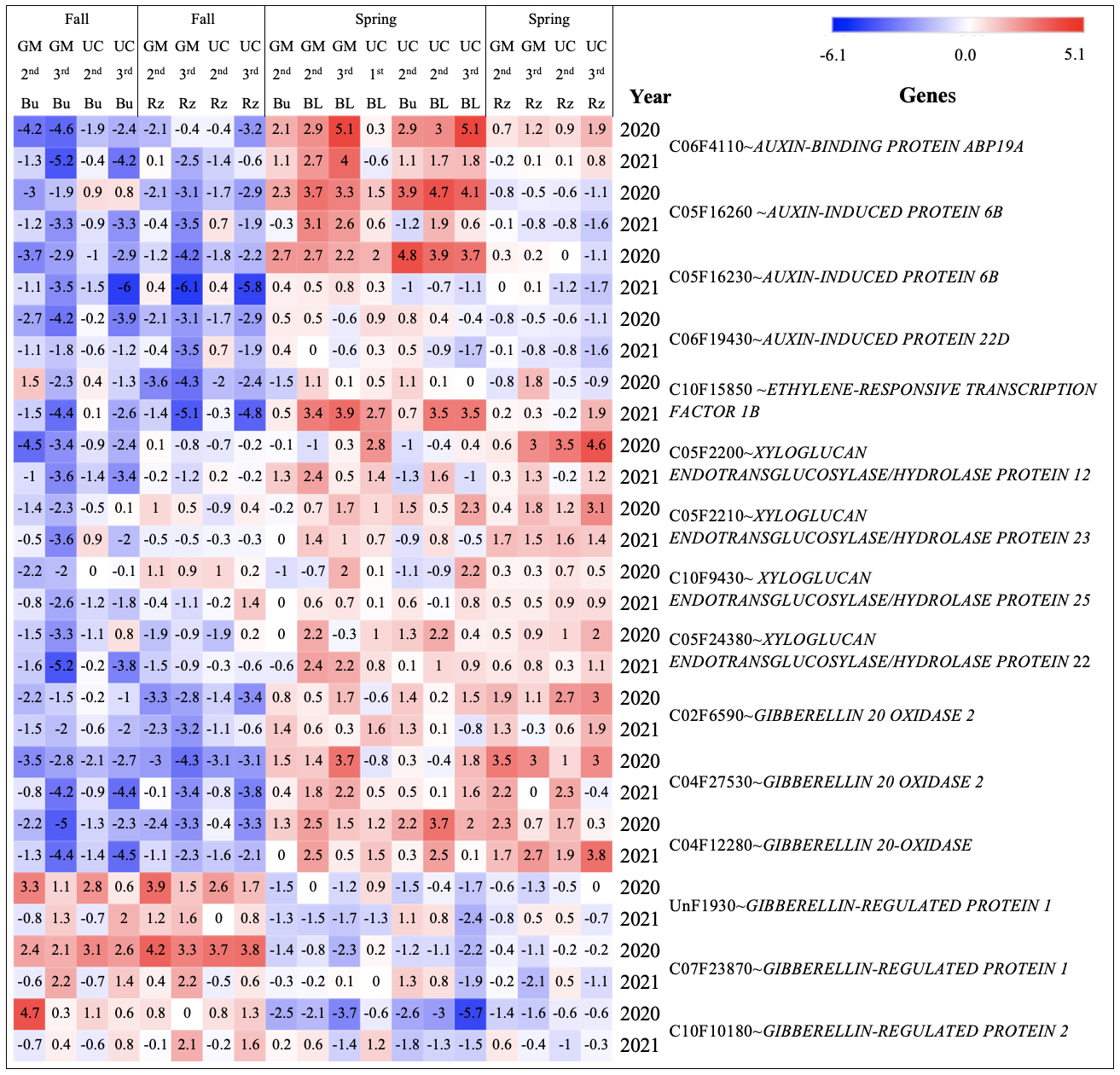


**Supplementary Figure 5** Expression patterns (log_2_fold differences) of differentially expressed genes involved in plant hormone signal transduction that showed expression patterns consistent with LT_50_ differences or similarities within dormant buds (Bu), growing buds (BL), and rhizomes (Rz) of asparagus cultivars ‘Guelph Millennium’ (GM) and ‘UC157’ (UC) during fall acclimation and spring deacclimation. Log_2_fold differences relative to dormant buds and rhizomes of first harvest are presented in the cells. Upregulated genes are color-coded red (log_2_fold difference > 0) and downregulated genes are color-coded blue (log_2_fold difference < 0). 1^st^, first harvest; 2^nd^, second harvest; 3^rd^, third harvest.

**
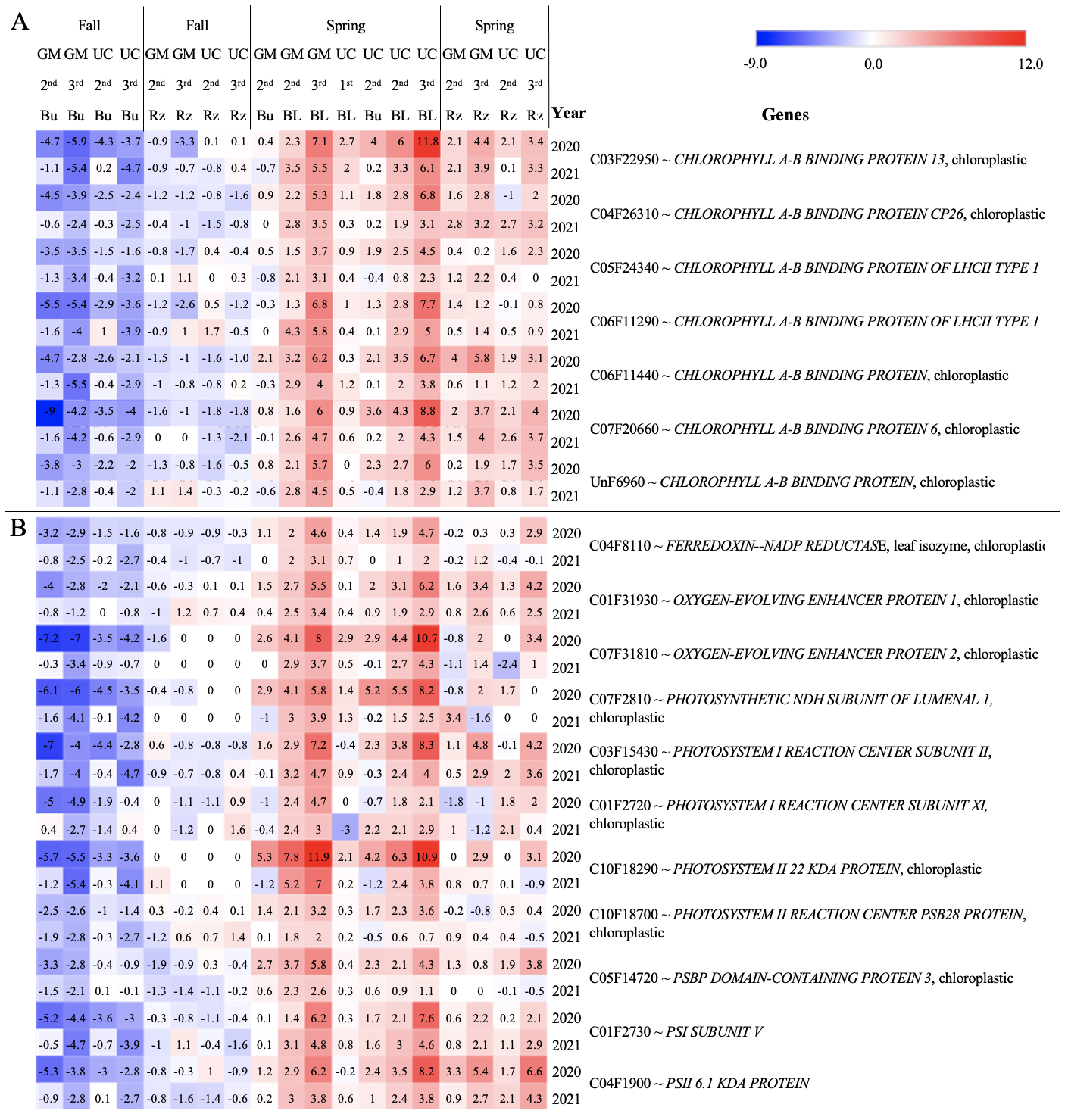
**

**Supplementary Figure 6** Expression patterns (log_2_fold differences) of differentially expressed genes involved in **(A)** photosynthesis-antenna proteins and **(B)** photosynthesis that showed expression patterns consistent with LT_50_ differences or similarities within dormant buds (Bu), growing buds (BL), and rhizomes (Rz) of asparagus cultivars ‘Guelph Millennium’ (GM) and ‘UC157’ (UC) during fall acclimation and spring deacclimation. Log_2_fold differences relative to dormant buds and rhizomes of first harvest are presented in the cells. Upregulated genes are color-coded red (log_2_fold difference > 0) and downregulated genes are color-coded blue (log_2_fold difference < 0). 1^st^, first harvest; 2^nd^, second harvest; 3^rd^, third harvest.

**
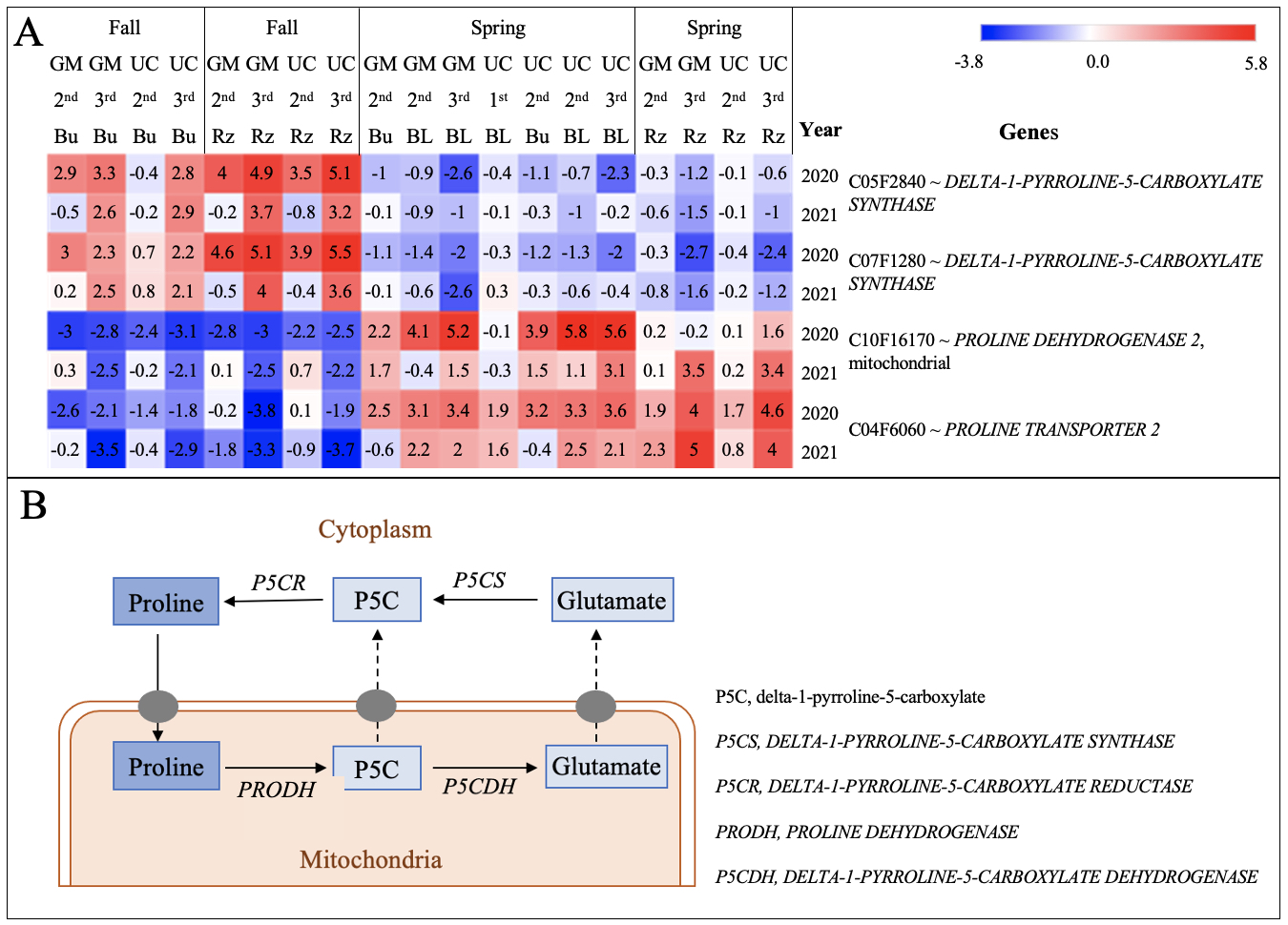
**

**Supplementary Figure 7 (A)** Expression patterns (log_2_fold differences) of differentially expressed genes involved in proline metabolism that showed expression patterns consistent with LT_50_ differences or similarities within dormant buds (Bu), growing buds (BL), and rhizomes (Rz) of asparagus cultivars ‘Guelph Millennium’ (GM) and ‘UC157’ (UC) during fall acclimation and spring deacclimation. **(B)** Proline metabolic pathways in asparagus and *A. thaliana*  to which differentially expressed genes were mapped. Pathway was modified from KEGG database (<http://www.kegg.jp>). Log_2_fold differences relative to dormant buds and rhizomes of first harvest are presented in the cells. Upregulated genes are color-coded red (log_2_fold difference > 0) and downregulated genes are color-coded blue (log_2_fold difference < 0). 1^st^, first harvest; 2^nd^, second harvest; 3^rd^, third harvest.

**
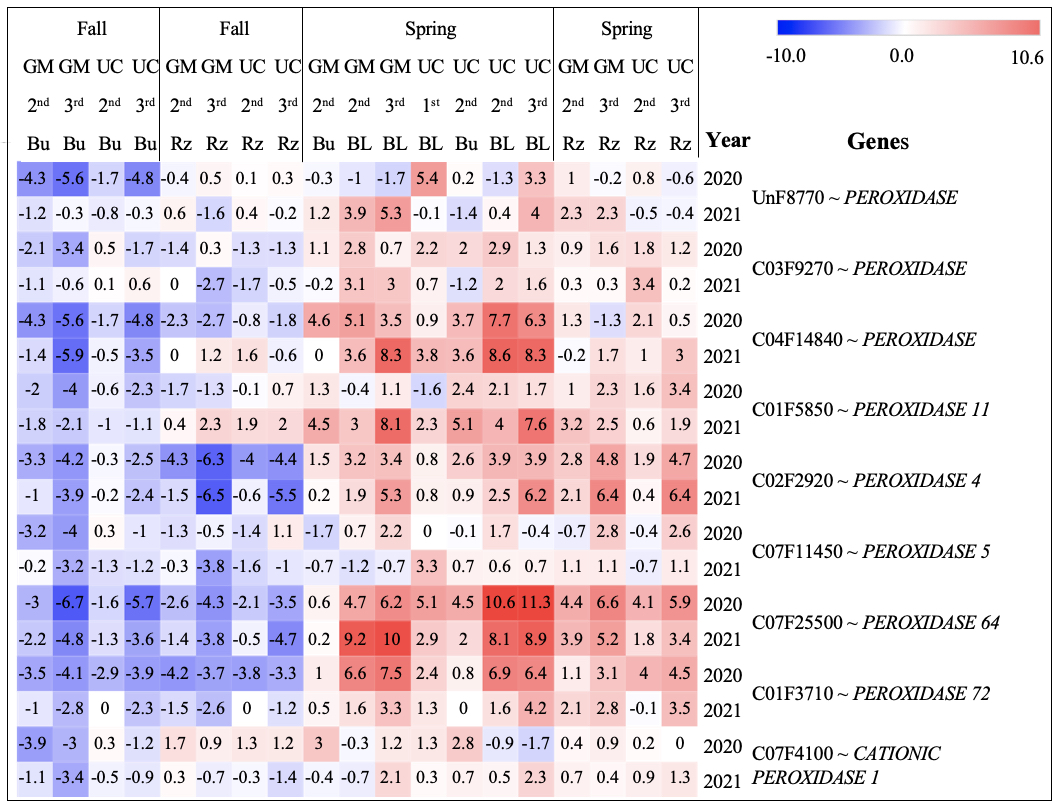
**

**Supplementary Figure 8** Expression patterns (log_2_fold differences) of differentially expressed genes involved in phenylpropanoid biosynthesis that showed expression patterns consistent with LT_50_ differences or similarities within dormant buds (Bu), growing buds (BL), and rhizomes (Rz) of asparagus cultivars ‘Guelph Millennium’ (GM) and ‘UC157’ (UC) during fall acclimation and spring deacclimation. Log_2_fold differences relative to dormant buds and rhizomes of first harvest are presented in the cells. Upregulated genes are color-coded red (log_2_fold difference > 0) and downregulated genes are color-coded blue (log_2_fold difference < 0). 1^st^, first harvest; 2^nd^, second harvest; 3^rd^, third harvest.


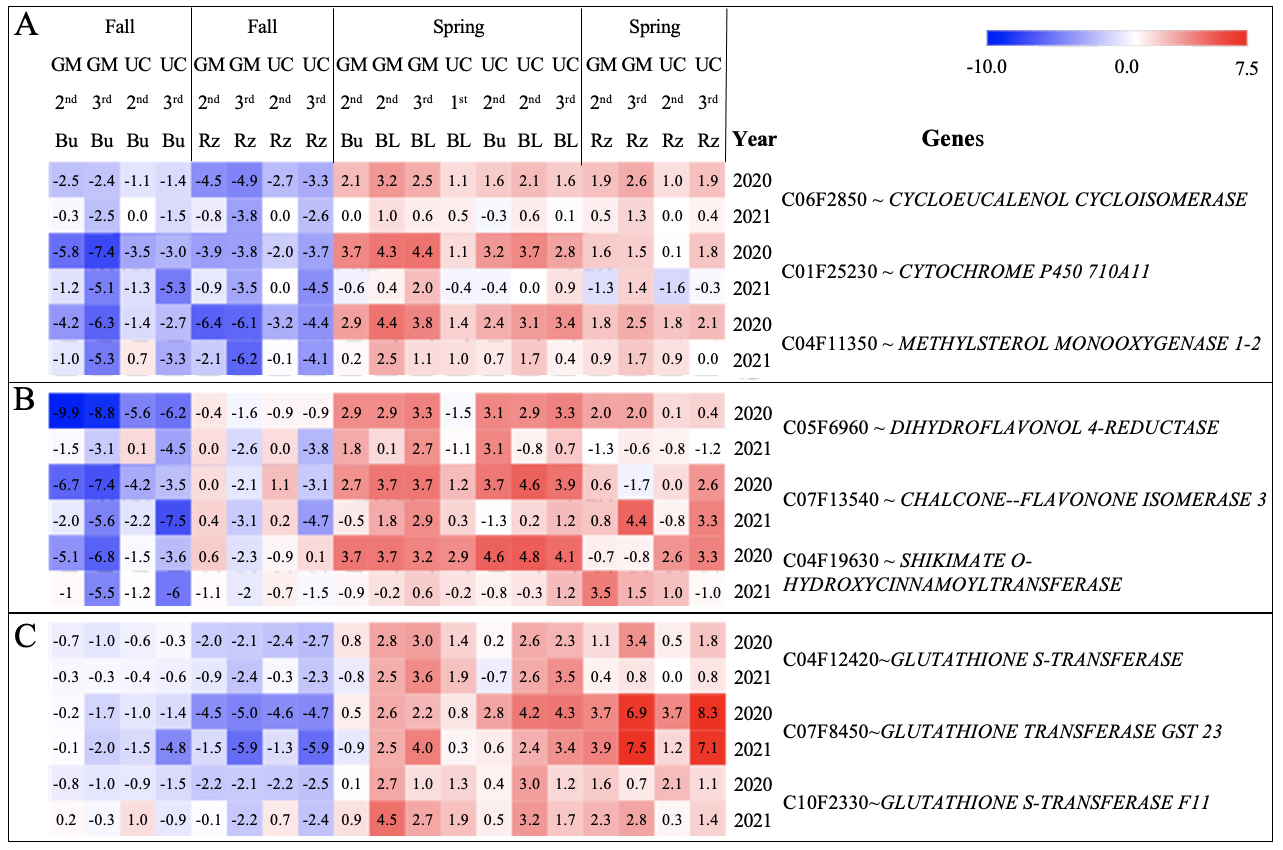


Supplementary Figure 9 Expression patterns (log_2_fold differences) of differentially expressed genes involved in (A) steroid biosynthesis, (B) flavonoid biosynthesis, and (C) glutathione metabolism that showed expression patterns consistent with LT_50_ differences or similarities within dormant buds (Bu), growing buds (BL), and rhizomes (Rz) of asparagus cultivars ‘Guelph Millennium’ (GM) and ‘UC157’ (UC) during fall acclimation and spring deacclimation. Log_2_fold differences relative to dormant buds and rhizomes of first harvest are presented in the cells. Upregulated genes are color-coded red (log_2_fold difference > 0) and downregulated genes are color-coded blue (log_2_fold difference < 0). 1^st^, first harvest; 2^nd^, second harvest; 3^rd^, third harvest.

**
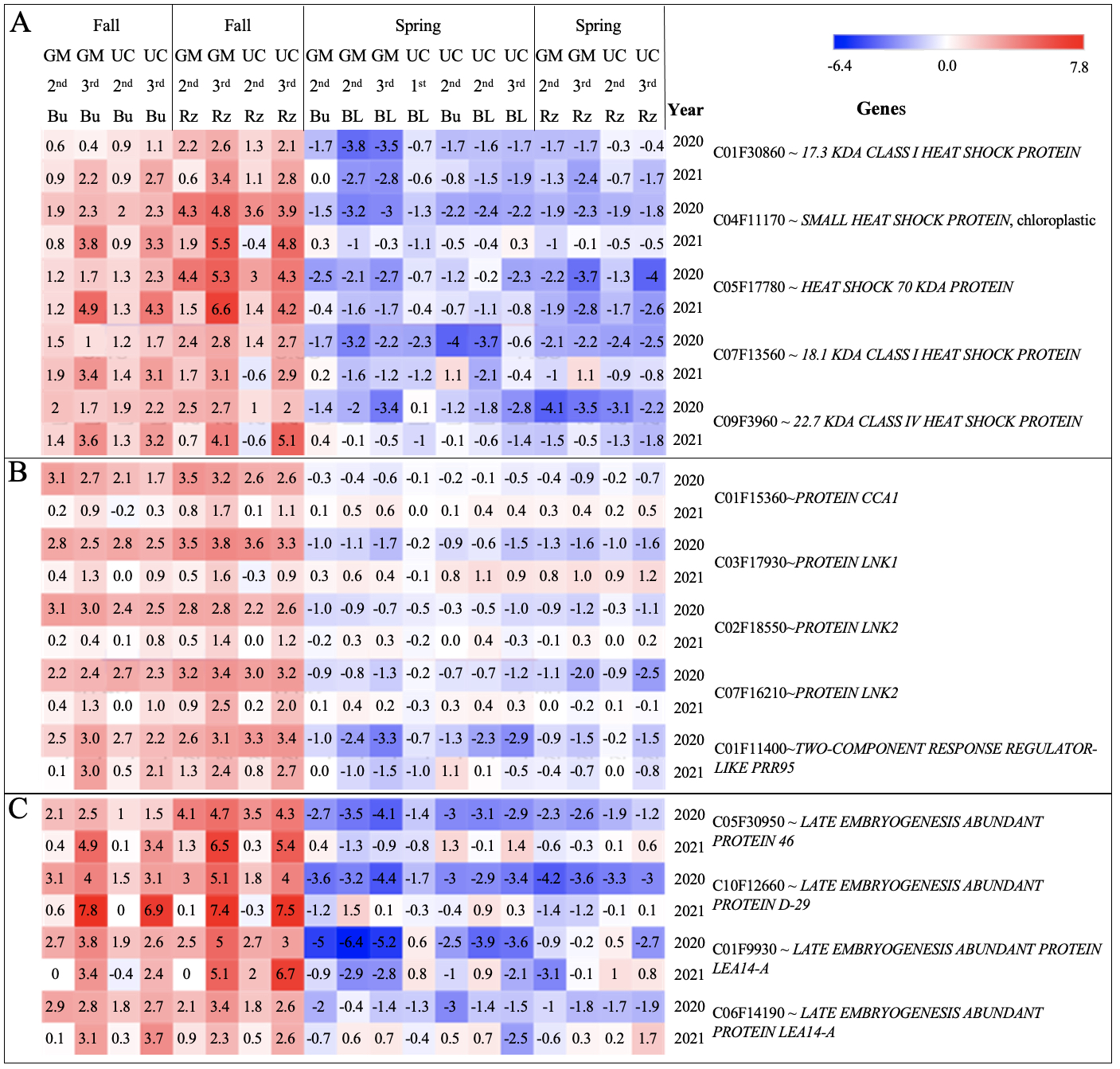
**

**Supplementary Figure 10** Expression patterns (log_2_fold differences) of differentially expressed genes involved in **(A)** protein processing in endoplasmic reticulum, **(B)** circadian rhythm, and **(C)** late embryogenesis abundant proteins that showed expression patterns consistent with LT_50_ differences or similarities within dormant buds (Bu), growing buds (BL), and rhizomes (Rz) of asparagus cultivars ‘Guelph Millennium’ (GM) and ‘UC157’ (UC) during fall acclimation and spring deacclimation. Log_2_fold differences relative to dormant buds and rhizomes of first harvest are presented in the cells. Upregulated genes are color-coded red (log_2_fold difference > 0) and downregulated genes are color-coded blue (log_2_fold difference < 0). 1^st^, first harvest; 2^nd^, second harvest; 3^rd^, third harvest.

**
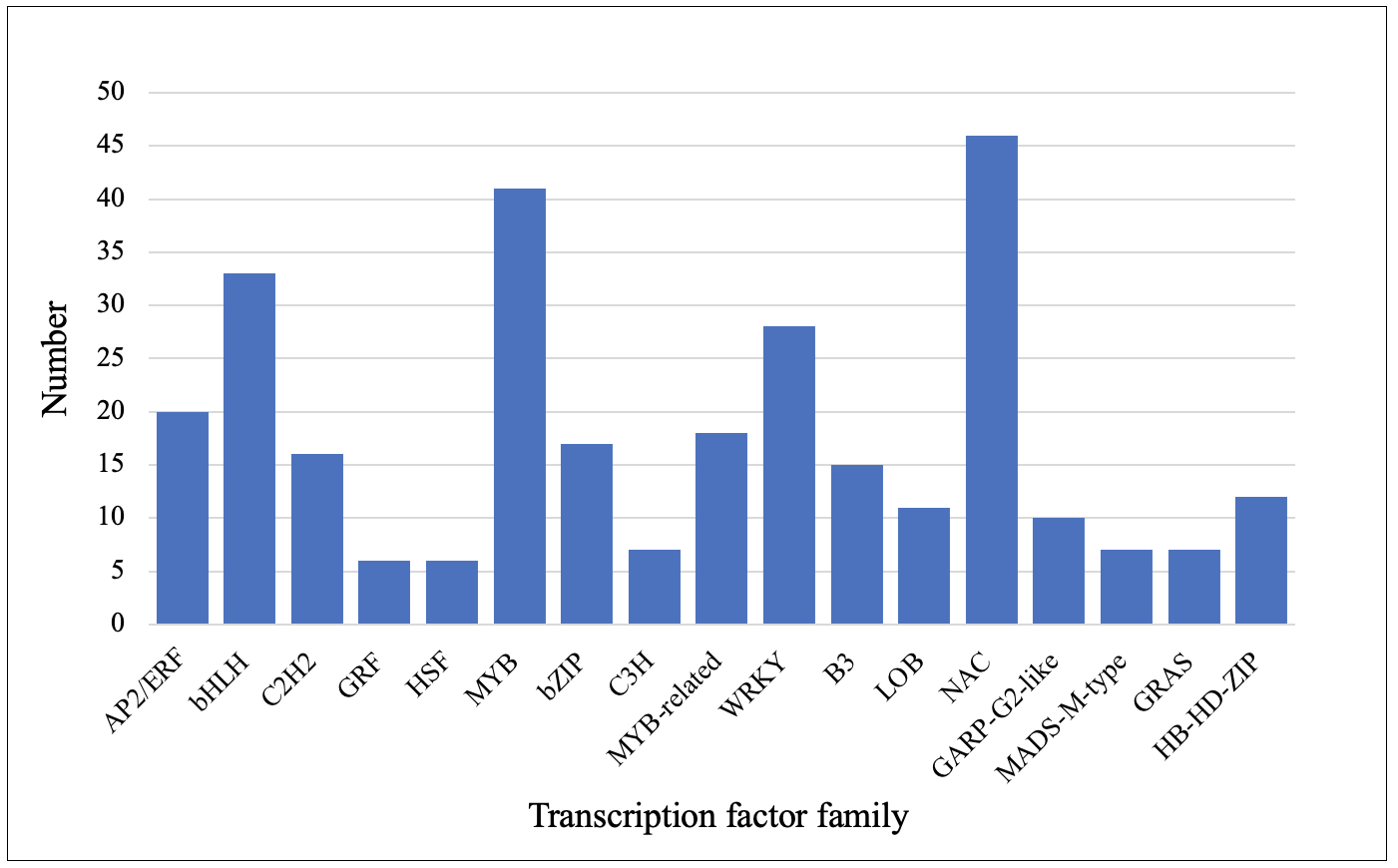
Supplementary Figure 11** Number and classification of transcription factor encoded genes differentially expressed within dormant buds, growing buds, and rhizomes of asparagus cultivars ‘Guelph Millennium’ (GM) and ‘UC157’ (UC) during fall acclimation and spring deacclimation in the RNA-Seq experiment.

**
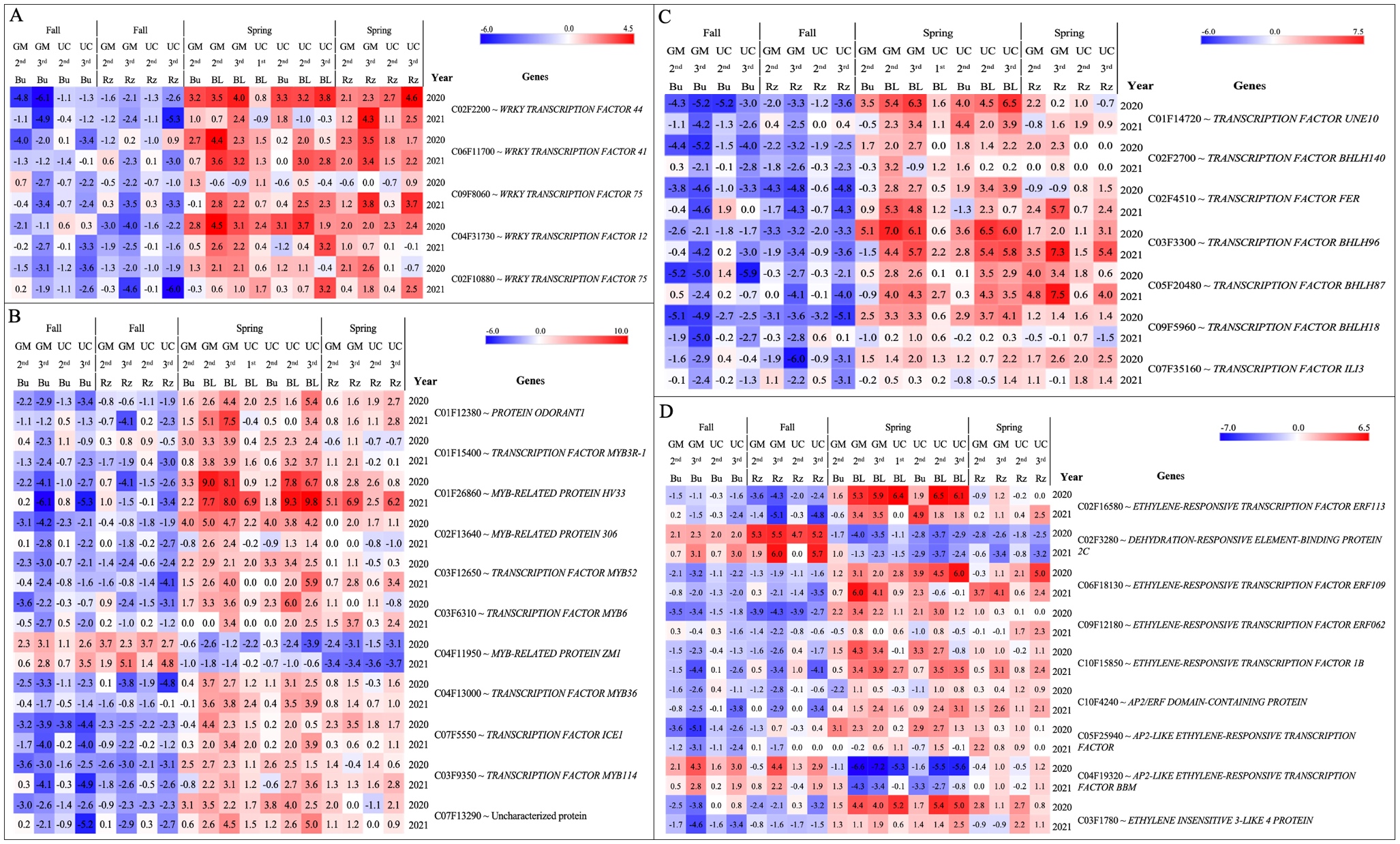
**

**Supplementary Figure 12** Expression patterns (log_2_fold differences) of **(A)** WRKY, **(B)** MYB, **(C)** basic helix-loop-helix (bHLH), and **(D)** APETALA2/ethylene response factor (AP2/ERF) transcription factors within dormant buds (Bu), growing buds (BL), and rhizomes (Rz) of asparagus cultivars ‘Guelph Millennium’ (GM) and ‘UC157’ (UC) during fall acclimation and spring deacclimation. Log_2_fold differences relative to dormant buds and rhizomes of first harvest are presented in the cells. Upregulated genes are color-coded red (log_2_fold difference > 0) and downregulated genes are color-coded blue (log_2_fold difference < 0). 1^st^, first harvest; 2^nd^, second harvest; 3^rd^, third harvest.


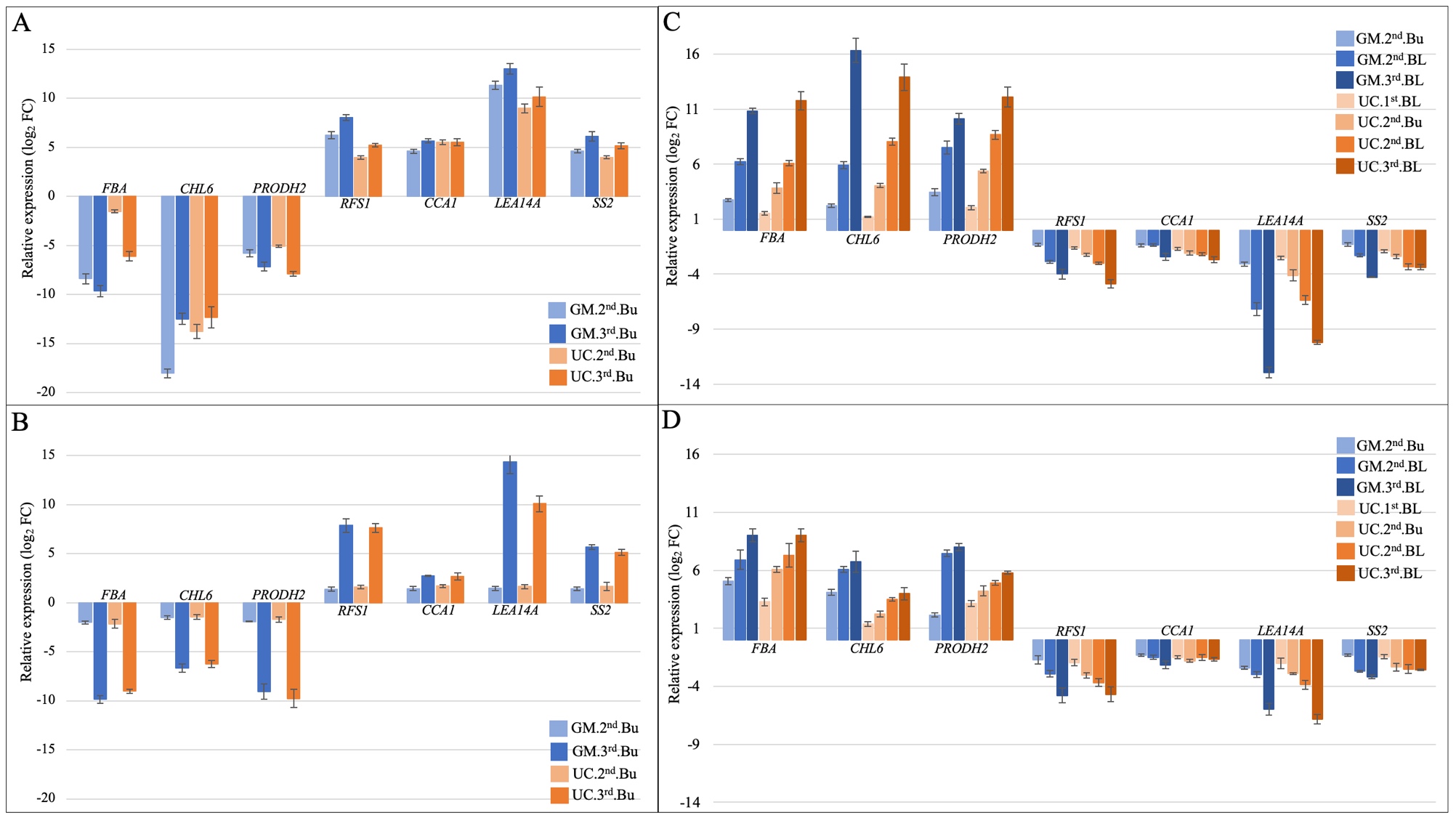


**Supplementary Figure 13** Relative gene expression (qRT-PCR) for validation of the seven selected genes in the dormant (Bu) and/or growing buds (BL) of asparagus cultivars ‘Guelph Millennium’ (GM) and ‘UC157’ (UC) during **(A)** fall 2020, **(B)** fall 2021, **(C)** spring 2020, and **(D)** spring 2021. Gene expression was relative in reference to the dormant buds of first harvest. Vertical bars are the standard error of means (n=3). *FBA, FRUCTOSE-BISPHOSPHATE ALDOLASE; CHL6, CHLOROPHYLL A-B BINDING PROTEIN 6; PRODH2, PROLINE DEHYDROGENASE 2; RFS1, GALACTINOL-SUCROSE GALACTOSYLTRANSFERASE 1; CCA1, CIRCADIAN CLOCK ASSOCIATED 1; LEA14A, LATE EMBRYOGENESIS ABUNDANT PROTEIN 14-A; SS2, SUCROSE SYNTHASE 2*. 1^st^, first harvest; 2^nd^, second harvest; 3^rd^, third harvest.


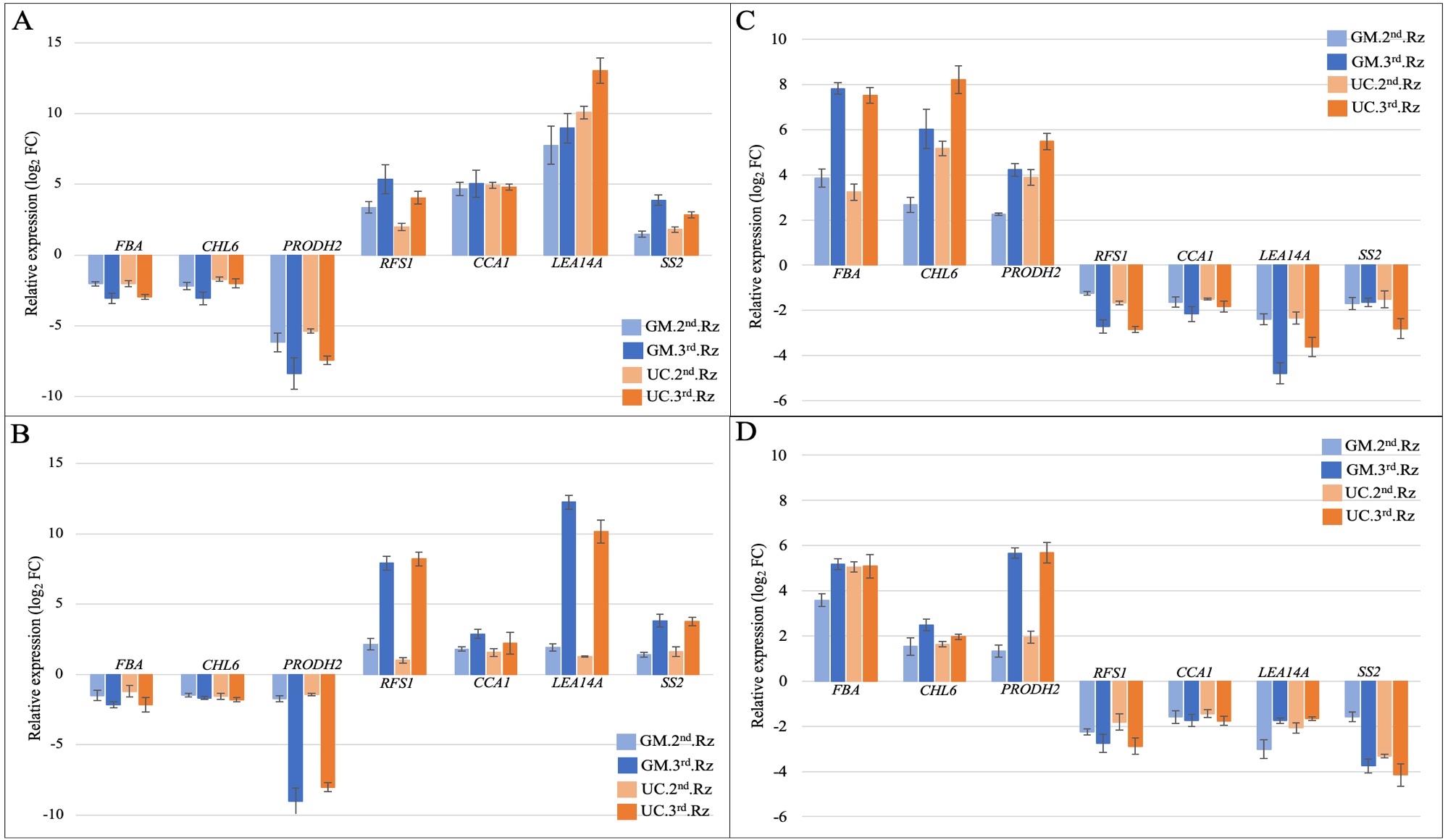


Supplementary Figure 14 Relative gene expression (qRT-PCR) for validation of the seven selected genes in the rhizomes (Rz) of asparagus cultivars ‘Guelph Millennium’ (GM) and ‘UC157’ (UC) during (A) fall 2020, (B) fall 2021, (C) spring 2020, and (D) spring 2021. Gene expression was relative in reference to the dormant buds of first harvest. Vertical bars are the standard error of means (n=3). *FBA, FRUCTOSE-BISPHOSPHATE ALDOLASE; CHL6, CHLOROPHYLL A-B BINDING PROTEIN 6; PRODH2, PROLINE DEHYDROGENASE 2; RFS1, GALACTINOL-SUCROSE GALACTOSYLTRANSFERASE 1; CCA1, CIRCADIAN CLOCK ASSOCIATED 1; LEA14A, LATE EMBRYOGENESIS ABUNDANT PROTEIN 14-A; SS2, SUCROSE SYNTHASE 2*. 1^st^, first harvest; 2^nd^, second harvest; 3^rd^, third harvest.

**
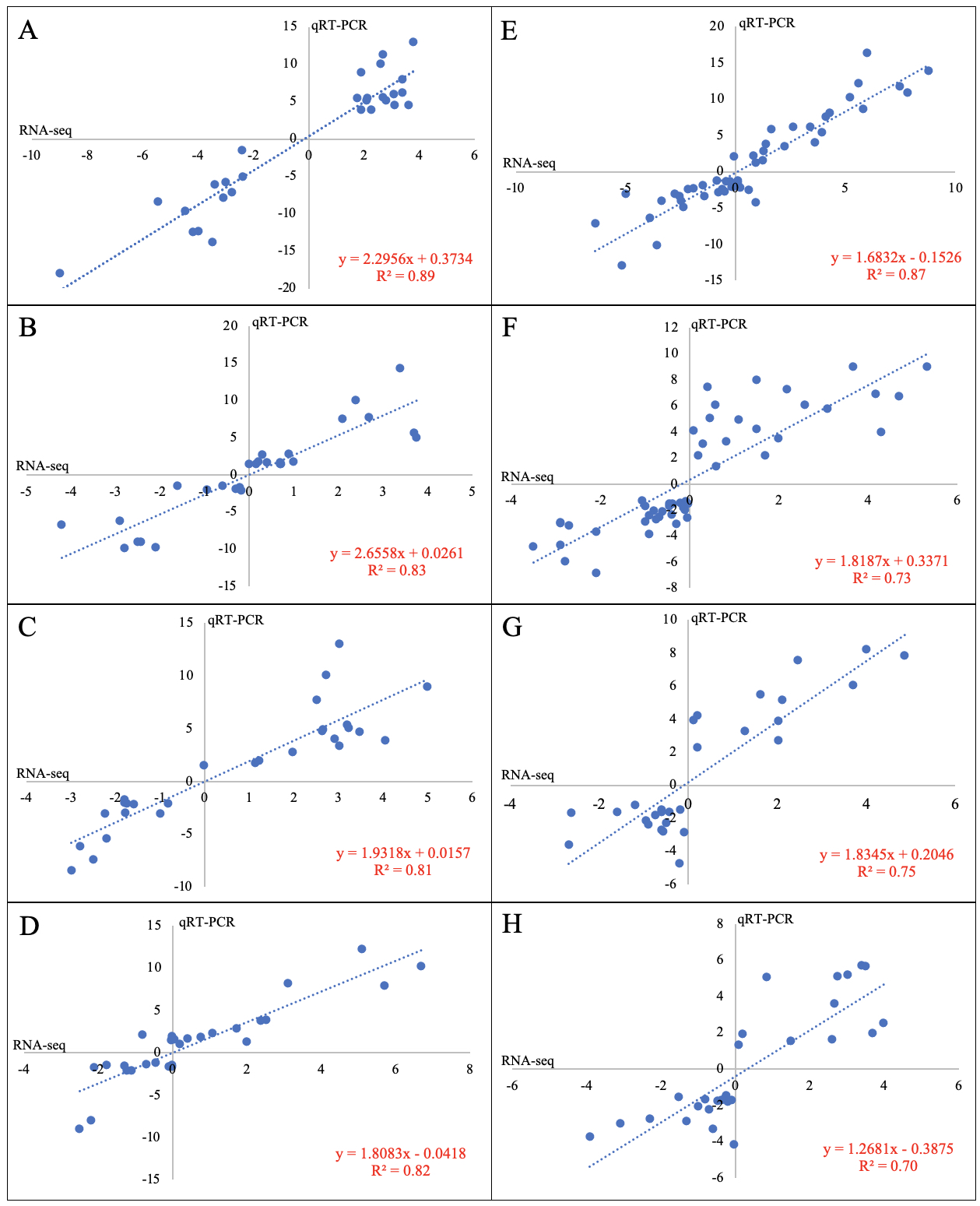
**

**Supplementary Figure 15** Linear regression analysis of gene expression patterns between RNA-Seq and qRT-PCR based on seven selected asparagus genes in the dormant buds during **(A)** fall 2020, **(B)** fall 2021, in the rhizomes during **(C)** fall 2020, **(D)** fall 2021, in the dormant and growing buds during **(E)** spring 2020, **(F)** spring 2021, and in the rhizomes during **(G)** spring 2020 and (**H)** spring 2021. Scatterplots were generated by the log_2_fold values from RNA-Seq data (X-axis) and relative expression values from qRT-PCR (Y-axis).
